# Supplementary material for: Estimation of total cardiovascular risk using the 2019 WHO CVD prediction charts and comparison of population-level costs based on alternative drug therapy guidelines: a population-based study of adults in Bangladesh
Source: BMJ Open. 2020 Jul 19;10(7):e035842. doi: 10.1136/bmjopen-2019-035842 (PMC7371224; doi:10.1136/bmjopen-2019-035842)
Supplement: Supplementary data [file bmjopen-2019-035842supp001.pdf]

Appendix Table 1: Cost of common drugs used to treat cardiovascular disease in Bangladesh (converted to US$)

Drug Name	Dose	Number of Tablets	Category	Median price (in Bangladeshi Taka)	Price (in US$)*
Aspirin	100 mg	100 tablets	Antiplatelet drugs	38BDT	0.45
Atenolol	50 mg	100 tablets	Antihypertensive, Beta-adrenoreceptor blocking drugs	77BDT	0.92
Captopril	25 mg	100 tablets	Antihypertensive, ACE inhibitors	300 BDT	3.58
Chrlorthalidone	25 mg	30 tablets	Antihypertensive, thiazide diuretics	60 BDT	0.72
Enalapril	10 mg	100 tablets	Antihypertensive, ACE inhibitors	200 BDT	2.38
Frusemide	40 mg	100 tablets	Antihypertensive, loop diuretics	53 BDT	0.63
Hydrochlorothiazide	25 mg	100 tablets	Antihypertensive, thiazide diuretics	70 BDT	0.83
Isosorbide dinitrate	10 mg	100 tablets	Angina treatment, nitrates - coronary vasodilators	35 BDT	0.42
Labetalol	100 mg	60 tablets	Antihypertensive, Beta-adrenoreceptor blocking drugs	361.2 BDT	4.31
Losartan	50 mg	100 tablets	Antihypertensive, Angiotensin-II receptor blocker	600 BDT	7.15
Lovastatine	20 mg	30 tablets	Lipid regulation, statin	303.3 BDT	3.62
Methyldopa	250 mg	100 tables	Antihypertensive, Centrally acting or central sympatholytic	308 BDT	3.67
Nifedipine (slow release)	20 mg	100 tablets	Angina treatment, calcium-channel blocker	64 BDT	0.76
Propranolol hydrochloride	10 mg	100 tablets	Antihypertensive, Beta-adrenoreceptor blocking drugs	24 BDT	0.29
Simvastatine	10 mg	30 tablets	Lipid regulation, statin	360 BDT	4.29

Spironolactone	25 mg	100 tablets	Antihypertensive, potassium-sparing diuretics & aldoesterone antagonist	202 BDT	2.41
Streptokinase	1.5 million unit/vial	One vial	Anticoagulant	3100 BDT	36.96
Warfarin	5 mg	100 tablets	Anticoagulant	300 BDT	3.58

Abbreviations: mg, milligrams; US, United States; BDT, Bangladeshi Taka

*Price conversion based on exchange rate on 02/07/2019
