# Supplementary material for: Estimation of total cardiovascular risk using the 2019 WHO CVD prediction charts and comparison of population-level costs based on alternative drug therapy guidelines: a population-based study of adults in Bangladesh
Source: BMJ Open. 2020 Jul 19;10(7):e035842. doi: 10.1136/bmjopen-2019-035842 (PMC7371224; doi:10.1136/bmjopen-2019-035842)
Supplement: Author's manuscript [file bmjopen-2019-035842.draft_revisions.pdf]

BMJ Open

BMJ Open is committed to open peer review. As part of this commitment we make the peer review history of every article we publish publicly available.

When an article is published we post the peer reviewers' comments and the authors' responses online. We also post the versions of the paper that were used during peer review. These are the versions that the peer review comments apply to.

The versions of the paper that follow are the versions that were submitted during the peer review process. They are not the versions of record or the final published versions. They should not be cited or distributed as the published version of this manuscript.

BMJ Open is an open access journal and the full, final, typeset and author-corrected version of record of the manuscript is available on our site with no access controls, subscription charges or pay-per-view fees (http://bmjopen.bmj.com).

If you have any questions on BMJ Open's open peer review process please email info.bmjopen@bmj.com

BMJ Open

Estimation of total cardiovascular risk using the WHO/ISH CVD prediction charts and comparison of population-level costs based on alternative drug therapy guidelines in Bangladesh

Journal:	*BMJ Open*
Manuscript ID	bmjopen-2019-035842
Article Type:	Original research
Date Submitted by the Author:	18-Nov-2019
Complete List of Authors:	Islam, Jessica; World Health Organization Country Office for Bangladesh, ; University of North Carolina at Chapel Hill Gillings School of Global Public Health, Department of Epidemiology<br>Zaman, MM; World Health Organization, Dhaka, Bangladesh,<br>Moniruzzaman, Mohammad; Shiga University of Medical Science, Public Health<br>Ara Shakoor, Shawkat; National Institute of Opthalmology<br>Hossain, A.H.M. Enayet; National Institute of Opthalmology
Keywords:	Cardiac Epidemiology < CARDIOLOGY, EPIDEMIOLOGY, Hypertension < CARDIOLOGY, Ischaemic heart disease < CARDIOLOGY, Myocardial infarction < CARDIOLOGY

SCHOLARONE™
Manuscripts

I, the Submitting Author has the right to grant and does grant on behalf of all authors of the Work (as defined in the below author licence), an exclusive licence and/or a non-exclusive licence for contributions from authors who are: i) UK Crown employees; ii) where BMJ has agreed a CC-BY licence shall apply, and/or iii) in accordance with the terms applicable for US Federal Government officers or employees acting as part of their official duties; on a worldwide, perpetual, irrevocable, royalty-free basis to BMJ Publishing Group Ltd ("BMJ") its licensees and where the relevant Journal is co-owned by BMJ to the co-owners of the Journal, to publish the Work in this journal and any other BMJ products and to exploit all rights, as set out in our licence.

The Submitting Author accepts and understands that any supply made under these terms is made by BMJ to the Submitting Author unless you are acting as an employee on behalf of your employer or a postgraduate student of an affiliated institution which is paying any applicable article publishing charge ("APC") for Open Access articles. Where the Submitting Author wishes to make the Work available on an Open Access basis (and intends to pay the relevant APC), the terms of reuse of such Open Access shall be governed by a Creative Commons licence – details of these licences and which Creative Commons licence will apply to this Work are set out in our licence referred to above.

Other than as permitted in any relevant BMJ Author's Self Archiving Policies, I confirm this Work has not been accepted for publication elsewhere, is not being considered for publication elsewhere and does not duplicate material already published. I confirm all authors consent to publication of this Work and authorise the granting of this licence.

For peer review only

Estimation of total cardiovascular risk using the WHO/ISH CVD prediction charts and comparison of population-level costs based on alternative drug therapy guidelines in Bangladesh

Running title: Total cardiovascular risk approach in Bangladesh

Authors: Jessica Yasmine Islam[1,2], M Mostafa Zaman[2,3], Mohammad Moniruzzaman[2,3], Shawkat Ara Shakoor[5], A.H.M. Enayet Hossain[5]

Author Affiliations:

[1]Department of Epidemiology, Gillings School of Global Public Health, University of North Carolina at Chapel Hill, North Carolina, USA

[2]Noncommunicable Disease Unit, World Health Organization, Country Office for Bangladesh, Dhaka, Bangladesh

[3]Shiga University of Medical Science, Public Health, Seta-tsukinowa-cho, Otsu, Shiga. 520-2192, Japan, Otsu, JP 520-2192

[3]Research and Publication Unit, World Health Organization Country Office for Bangladesh, Dhaka, Bangladesh

[5]National Institute of Ophthalmology. Sher-e-Bangla Nagar, Dhaka, Bangladesh

Corresponding Author:

Jessica Yasmine Islam, PhD, MPH
The University of North Carolina at Chapel Hill
Gillings School of Global Public Health
Department of Epidemiology
Chapel Hill, NC, USA 27599-7400
Email: islamjy@email.unc.edu

Manuscript word count: 3869

For peer review only

For peer review only - http://bmjopen.bmj.com/site/about/guidelines.xhtml

Abstract

Objective: The objective this study is to apply estimate the population distribution of 10-year cardiovascular disease (CVD) risk among Bangladeshi adults aged 40 years and above, using the WHO recommended WHO/ISH CVD prediction charts. Additionally, we compared the cost of pharmacologic treatment based on CVD risk (cardiovascular risk threshold ≥ 30%/ ≥ 20%) to single risk factor (hypertension) cutoff levels for pharmacologic intervention in Bangladesh.

Study Design: Cross-sectional, population-based study

Setting and Participants: In 2013, we collected data from a nationally representative cross-sectional study of adults aged ≥40 years from urban and rural areas of Bangladesh (n = 6189). Using the World Health Organization / International Society of Hypertension (WHO/ISH) risk prediction charts, CVD risk was calculated and categorized as low(<10%), moderate(10-<20%), high(20-<30%), and very high risk(≥30%). We estimated drug therapy costs using the lowest price of each drug class available(aspirin, thiazide diuretics, statins, and angiotensin-converting enzyme inhibitors). We compared the total cost of drug therapy using the total risk vs. single risk factor approach.

Primary Outcome Measures: Our primary outcome was 10-year CVD risk categorized as low(<10%), moderate(10-<20%), high(20-<30%), and very high risk(≥30%).

Results: The majority of adults (85.2%, 95% CI: 84.3–86.1) have a 10-year risk of CVD of less than 10%. The proportion of adults with a 10-year CVD risk of ≥20% and ≥30% was 1.8% and 0.6%, respectively. Using the total risk approach would reduce drug costs per million population

For peer review only - http://bmjopen.bmj.com/site/about/guidelines.xhtml

to $635,976 (risk of ≥ 20%) or $173,448 (≥ 30%) for CVD treatment and prevention in comparison to using the single risk factor approach ($5,665,968).

Conclusion: To reduce health care expenditure for the prevention and treatment of CVD in Bangladesh, a total risk approach using the WHO/ISH risk prediction chart may lead to cost-savings and potentially improved treatment coverage.

Keywords: non-communicable diseases, Bangladesh, cardiovascular disease, pharmacologic intervention, cost, chronic disease

For peer review only

Article Summary

Strengths and Limitations of the Study

- This study utilized a multistage, geographically clustered, probability-based sampling approach to produce nationally representative data for Bangladesh of adults aged 40 years and above.
- Currently, Bangladesh has not incorporated clinical guidelines for screening or treatment of risk factors based on absolute CVD risk scores, or estimation of the population distribution of CVD risk over time. This study provides evidence for incorporating the WHO/ISH CVD Risk Prediction Charts and potential cost-savings of strategy implementation.
- The WHO/ISH CVD Risk Prediction Charts should be applied to a population who have not experienced a CVD event in the past. We were unable to confirm the medical history of participants using medical charts or health records and relied on self-report, leading to the potential for measurement error and recall bias.
- Our cost estimates were based on the prevalence of each risk approach in our study sample. Although we present the total number of people estimated to require drug treatment using 2016 census data, we were unable to identify population estimates of only those at risk of their first CVD event due to lack of surveillance data.

For peer review only

For peer review only - http://bmjopen.bmj.com/site/about/guidelines.xhtml

Background

Globally, cardiovascular disease (CVD) is the leading cause of death and disproportionately impacts low- and middle-income countries (LMICs), where over 75% of CVD-related deaths occur [1]. People living in LMICs are at high risk of developing CVD due to the absence of integrated primary care for early detection and prevention of CVD-related risk factors. Limited access to primary care and the growing burden of CVDs is a significant cause of poverty in LMICs and hinders the macroeconomic development of many countries[2]. LMICs are estimated to experience cumulative economic losses exceeding 7 trillion US dollars over the next 15 years due to morbidity and mortality caused by noncommunicable diseases, including CVD[3]. As such, the significance of the CVD epidemic has gained increasing international recognition over the past decade, leading to the development of several international guidelines for CVD control and prevention[4].

In 2007, the World Health Organization (WHO) published pocket guidelines, including CVD risk prediction charts, designed for health care workers in LMICs to guide patient 10-year risk stratification for heart attack or stroke. There are two possible strategies suitable for a low resource setting to assess the risk of a cardiovascular event and identify those at high risk of a fatal CVD outcome: 1. Utilize a single risk factor management strategy, which focuses on one condition at a time, such as hypertension; 2. Utilize a more holistic approach considering several risk factors such as age, tobacco use, gender, diabetes diagnosis, blood pressure, and blood cholesterol when measured. Through the total risk approach, pocket guidelines help to identify high-risk patients that are in imminent danger of a heart attack or stroke for timely pharmacologic treatment or surgical interventions. Currently, individuals in LMIC's, including Bangladesh, are often offered pharmacologic interventions based on the presence of single CVD risk factors, such as high blood pressure. However, the single risk factor approach can result in overtreatment or neglecting to treat those with an overall higher CVD-risk based on CVD risk factors. Applying the total risk approach via the WHO/ISH prediction charts in a

For peer review only - http://bmjopen.bmj.com/site/about/guidelines.xhtml

nationally representative sample may provide an opportunity to estimate population-level distribution of CVD risk and inform CVD treatment policy recommendations [5,6].

Currently, Bangladesh has not incorporated clinical guidelines for screening or treatment of risk factors based on absolute CVD risk scores, or estimation of the population distribution of CVD risk over time. Data are needed to support the implementation of the WHO/ISH prediction charts among the Bangladeshi population and demonstrate the benefit of the WHO recommendation on CVD prevention. Prior studies conducted in Bangladesh have estimated CVD risk among adults residing in rural areas only and have not included a nationally representative population [7-9]. Additionally, no prior studies have estimated the potential costs of pharmacological treatment for CVD in Bangladesh using either the single risk factor or total risk approach. As such, our objective was to assess the distribution of absolute CVD risk among a nationally representative sample of Bangladeshi adults using the WHO/ISH risk prediction chart recommended for the WHO South and South-East Asian Region (SEARO). We also compared the costs of drug treatments for CVD prevention using the total cardiovascular risk thresholds (≥20% and ≥30%) and with single risk factor cutoff levels (blood pressure ≥ 140/90 mm Hg).

Methods

<u>Study design and setting</u>

Data for the current study were analyzed from a population-based cross-sectional study conducted from September to December 2013 to assess the burden of blindness and low vision among adults (men and women) aged 40 years and above in Bangladesh. The target population of this survey included men and women residing in Bangladesh over the age of 40 years. The exclusion criteria included tourists and the institutionalized, such as residents of a military base, hospital, prisons, nursing homes, and other such institutions. We obtained ethical approval for this study from the Institutional Review Board of the National Institute of Ophthalmology. We provided participants with detailed study information using a printed handout prepared in

For peer review only - http://bmjopen.bmj.com/site/about/guidelines.xhtml

Bengali to inform them of the objective of the study. Written consent was obtained from participants through signature or, if not possible, through thumbprint.

Sampling Frame

We adopted a multistage, geographically clustered, probability-based sampling approach to obtain a nationally representative sample of Bangladesh, as previously described [10-12]. Population statistics were obtained using the 2011 national census conducted by the Bangladesh Bureau of Statistics (BBS) to create the sample frame [13]. The sampling frame included 64,407 primary sampling units (PSUs) covering all seven divisions of the country. We randomly selected seventy-two PSUs (25 urban and 47 rural) from seven divisions, with the probability of selection proportional to the population size of each division. In each PSU, we selected 100 consecutive households as the secondary sampling unit.

For each household, a trained field data collector approached the head of the household or the family member most knowledgeable of the residents to screen for eligible participants. The respondent for screening was asked to describe the composition of household residents who considered the home to be their primary place of residence as of the night before. A list was composed and ordered from youngest to oldest age in years starting from 40 years. Using the list of eligible residents, we used the Kish table approach to randomly select one respondent from each home. The respondent was asked to come to a nearby health center the next day to administer the survey by trained study interviewers and undergo the relevant medical examination by the study physician. Based on the medical review, participants were followed-up with by the providers at the health center for treatment, when appropriate.

Patient and public involvement

There was no patient or public involvement in the implementation of this study or interpretation of analytic results.

For peer review only - http://bmjopen.bmj.com/site/about/guidelines.xhtml

Data Collection

To ensure effective and uniform data collection, field interviewers underwent a 7-day training on the interview process and methodology by the study ophthalmologists and epidemiologists. The training included an in-depth review of the survey content and protocol for completing the demographic information interview schedule questionnaire (a modified WHO/PBL Version III). We provided a detailed survey protocol manual outlining the survey activities, the questionnaire interview, and information about the duties and responsibilities of all survey personnel to each member of the data collection team.

Demographic data were obtained, including age, sex, marital status, educational level, and occupation, using a structured questionnaire survey. Data regarding tobacco use, health history, and treatment history were also collected. Participants were asked if they smoked (e.g. cigarette, hookah, pipe) or if they used smokeless tobacco (e.g. chewing tobacco, jorda, white lead) to assess history of tobacco use. Medical history for a prior diagnosis of high blood pressure or hypertension, diabetes, renal disease, or any cardiovascular diseases by a health care provider was obtained from each participant. Medication history was obtained based on self-report, including medication for high blood pressure, diabetes, malaria, steroids, tuberculosis, and among women, history of oral contraception. The questionnaire was translated from English to Bengali, adapted, and validated before data collection.

Physical measurements, including height, weight, and blood pressure were collected. BP was measured by an appropriately calibrated aneroid sphygmomanometer by a trained field interviewer using appropriately sized arm cuffs. BP measurements were consistently taken on the right arm at the level of the heart and elbow-assisted while the participant was seated. The initial measurement was performed after five minutes of rest on the right arm. After two minutes, the second measurement was taken. The mean of these two BP readings was utilized as the final BP for each participation. To measure blood glucose levels, we obtained random blood glucose samples [14]. Capillary blood samples were consistently taken using the right arm and

For peer review only - http://bmjopen.bmj.com/site/about/guidelines.xhtml

index finger with a glucometer, namely Accu-chek Advantage (Roche Diagnostics Division, Grenzacherstrasse, Switzerland).

Estimation of 10-year CVD risk

The WHO/ISH prediction charts were used to grade cardiovascular risk[15]. The charts developed by the WHO/ISH provide the 10-year risk of a fatal or non-fatal major cardiovascular event, such as myocardial infarction or stroke, based on age, sex, blood pressure, smoking status, total blood cholesterol, and the presence or absence of diabetes mellitus for 14 WHO epidemiological sub-regions. Two sets of charts are available: one set is used in settings where blood cholesterol can be measured, and the other set is suitable for when blood cholesterol has not been measured. We utilized the South-East Asian Region (SEAR) D WHO/ISH prediction chart where total blood cholesterol cannot be measured to estimate the 10-year risk of a CVD event among Bangladeshi adults,

The prediction chart grades cardiovascular risk using the following categories: age (1: 40–49 years; 2: 50–59 years; 3: 60–69 years; 4: 70 years and older), sex (men and women), smoking (never smoker, current smoker or ex-smoker), systolic blood pressure (<140 mmHg, 140 to <160, 160 - <180, and ≥180), and the presence or absence of diabetes. The risk categories for 10-year combined acute myocardial infarction and stroke (fatal and non-fatal) are as follows: 10%, 10 - <20%, 20 to <30%, 30 to <40, and ≥ 40%. For this analysis, we combined the last two categories of risk as ≥30%.

Observations with missing values were dropped from the analysis. We did not anticipate any bias from the complete-case analysis approach as the number of missing observations for key variables was less than 2%: smoking status, n=18 or 0.3% missing values; systolic blood pressure, n = 22 or 0.4% missing values; diastolic blood pressure, n = 25 or 0.4% missing values; and blood glucose levels, n = 7 or 0.1%;

Data Analysis

For peer review only - http://bmjopen.bmj.com/site/about/guidelines.xhtml

We present sociodemographic variables using mean (standard deviation) or the median (interquartile range) for continuous variables and proportion for categorical variables. We conducted bivariate analyses by sex and age group. We used the chi-square test to assess for any significant differences in CVD risk distribution across sex. For estimating the cost of medicines per million per year (population aged 40 years or older), we used the lowest price of each drug class available in the market (generic preparation of aspirin, thiazide diuretics, statins, and angiotensin-converting enzyme inhibitors). Appendix 1 includes further details regarding the specific costs of common drugs used to treat cardiovascular disease in Bangladesh.

To calculate costs using the single risk factor approach, we included all people with BP ≥140/90. Similarly, to calculate costs based on the cardiovascular risk approach, we included the following categories: (1) people with high cardiovascular risk (≥30% and ≥20%), who are recommended four drugs [6]; and (2) people with BP ≥140/90, who are recommended antihypertensive treatment. To calculate the estimated annual total cost of CVD medication treatment per million population (aged 40 years or older), we multiplied the percentage of the population at risk and the price of medicine in Bangladesh. We included an estimate of the total number of people estimated to require drug treatment we multiplied the prevalence of the population requiring medication based on each approach based on our study estimates, by the number of people in the general population in 2016 [16] stratified by gender and age group.

Results

<u>Demographic Characteristics</u>

The mean age of included participants was 52.9 years (men: 53.5 years, women: 52.5 years) (Table 1). The average level of educational attainment was 3.1 years of education; women were generally less educated than men (2.1 years vs. 4.3 years, respectively). The majority (80%) of women were housewives, and among men, the most common occupation was

For peer review only - http://bmjopen.bmj.com/site/about/guidelines.xhtml

an industrial worker or day laborer (51.2%). Overall, over one-third of participants ever used smoking tobacco, and over half ever used smokeless tobacco. Few participants drank alcohol in the past 30 days (1.2%). The mean BMI was 21.9 kg/m^{2,} and the mean waist circumference was 82.4 cm.

Distribution of Cardiovascular Risk

We summarized the distribution of cardiovascular risk in the population overall and stratified by sex in Table 2. Over 85% of participants had a low (< 10%) 10-year cardiovascular risk, and this proportion was significantly different across sex (p<0.001). Almost all (97.7%) of the study population were categorized as having cardiovascular risk < 20%. A higher proportion of men (2.2%) were categorized as high risk than women (1.4%) (p = 0.029). Overall, very few participants (< 1%) were categorized as very high risk or with a cardiovascular risk of ≥ 30%.

Cost of Drug Treatment

If a ≥30% threshold of CV risk alone was applied for drug treatment, 0.56% of the total population requires drug treatment (0.75% in urban areas, 0.47% in rural areas). Lowering the cardiovascular risk threshold (from 30% to 20%) alone increased the number of people requiring treatment by almost five times (0.56% [34 of 6090] to 2.3% [140 of 6090]) (Table 3). The consequence was a substantial increase in health care expenditure, as described in Table 4. Conversely, if a single risk factor approach was applied, and all those with hypertension (a persistent SBP ≥140 and/or DBP ≥90) were treated, about 19.6% (1193 of 8,625) of the sample would require drug treatment, specifically antihypertensives; more than 10 times the proportion identified when using the total cardiovascular risk approach alone. Including individuals with raised BP (≥160/100 mm Hg) but with a 10-year CVD risk < 30% or < 20% would increase the percentage of people requiring drug treatment from 0.56 and 2.3, to 4.5% and 3.8%, respectively. This proportion was about three times lower than the proportion of participants using the single risk factor approach (Table 3).

For peer review only - http://bmjopen.bmj.com/site/about/guidelines.xhtml

Comparison of Cost by Approach

Next, we compared the estimated annual cost of medicines per million population for implementing the total risk approach vs. the single risk factor approach. Table 4 shows the estimated number of people aged 40 years or older requiring drug treatment stratified by age group and gender. The estimate showed that if the single risk factor approach is applied in Bangladesh with its percentage of population at risk and the lowest price of medicine in the country, the cost per million population (aged 40 years or older) of treating those with BP ≥140/90 would be $5,665,968.0 US$; if the absolute risk approach were applied, the cost of treating those with 10-year risk of CVD ≥30% per million population (aged 40 years or older) would be $635,976.0, almost nine times less (Figure 1). The cost estimation was based on the percentage of the population at different levels of risk and the differences in the price of generic medicines. For this analysis, we focused on the cost of pharmacological treatment as it is the most critical contributor to the overall direct costs of CVD treatment in Bangladesh. We assumed that other costs of CVD treatment and prevention service delivery, such as health facilities and wages of health workers, are similar for both approaches of service delivery[6].

Discussion

Using this nationally representative survey of Bangladeshi adults aged 40 years and above, we found that the majority of adults (97%) were at a low or moderate ten-year risk of myocardial infarction and stroke. The proportion of adults requiring drug treatment rose from 0.56% to 2.3% when the threshold of CVD risk was changed from ≥30% to ≥20%, respectively; which was lower the proportion than the single risk factor approach (19.6%). Our data demonstrated that using a single risk factor approach to manage individual cardiovascular risk factors is costlier ($5,665,968 per million population) than using the total risk approach (CVD risk ≥30, $173,448 per million population; CVD risk ≥20, $635,976 per million population), as a

For peer review only - http://bmjopen.bmj.com/site/about/guidelines.xhtml

more substantial proportion of adults will need drug treatment. Findings from this analysis support the implementation of clinical guidelines using CVD risk scores calculated using the WHO/ISH prediction charts to appropriately identify patients at highest risk of CVD development over ten years in Bangladesh.

In our study using nationally representative data, we found that the 10-year risk of CVD was low (<10%) among the vast majority of participants (85.2%). Additionally, only 2.4% of adults were at high risk (≥ 20%) of a CVD event within the next ten years. Two prior studies have reported absolute CVD risk among the Bangladeshi population (using the WHO/ISH Tool or any other absolute risk scoring system)[8 17]. In contrast to our study, Fatema et al. found that 10% of Bangladeshi adults living in rural areas were at high risk (≥ 20%) of a CVD event within the next ten years, and half of these adults fell in the very high-risk category (≥ 30%). These proportions were the same when total cholesterol was incorporated in the WHO/ISH CVD Risk-Assessment Tool. Unfortunately, we were unable to measure total blood cholesterol and were unable to make similar comparisons. In another rural Bangladeshi population, the proportion of participants at a high-risk (≥ 20%) of a CVD event in 10 years was 2.1%, which is similar in proportion to our finding[17]. Differences in CVD risk may be attributable to environmental, sociodemographic, and lifestyle factors. No other studies have been conducted to assess the 10-year risk of CVD using the WHO/ISH tool. Here, we present novel data using a nationally representative sample, which may be generalizable to the population of Bangladesh. Data we present may be used to inform policymakers decisions on clinical guidelines and resource allocation for treatment of CVDs in Bangladesh.

Our results demonstrate that using a single risk factor approach to manage individual CVD risk factors to prevent heart attacks and strokes would cost more than when using the total risk approach at either the 20% or 30% threshold due to higher drug costs. In Bangladesh, about 60% of out-of-pocket expenditure patients face goes towards drugs directly bought from pharmacies, diagnostics, and informal providers[18]. Additionally, patients in Bangladesh

For peer review only - http://bmjopen.bmj.com/site/about/guidelines.xhtml

experience a high level of uncertainty regarding out-of-pocket costs due to unforeseen costs on diagnostics and medications, including under-the-table payments to providers for reliable and timely services[19]. The cost of treatment for CVD frequently leads to catastrophic health expenditure and impoverishment as a result of use of health services; the proportion of catastrophic spending for treatment is highest among those from the lowest quintiles of wealth (14%) compared to those with high wealth and high socioeconomic status (6.6%) [20]. The economic impact of the sequelae of CVD is particularly severe in Bangladesh where the out-of-pocket expenditures for chronic disease management are high.

In addition to benefits to the patient, implementing the total risk approach would also be beneficial to the health care system in Bangladesh by improving NCD preventive service delivery and the use of guidelines for adequate care. Currently, Bangladesh is categorized by the World Bank as a lower-middle-income country with emerging health challenges as the burden of NCDs continues to grow. In 2015, an estimated 67% of all deaths in Bangladesh were due to NCDs and the risk of premature death from chronic disease among adults aged 30-70 years was 22%[21]. Indeed, CVDs and circulatory diseases are the leading cause of mortality and morbidity in Bangladesh. Despite this substantial burden, preventive services for CVDs in Bangladesh are limited. In 2014, an estimated 16% of health care facilities across the country (i.e. hospitals, community clinics) had the resources to diagnose, prescribe treatment for, and manage patients with CVDs[22]. Among facilities with the capacity to offer services for CVD management, about only 20% utilized established guidelines for hypertension treatment and less than one-third had essential CVD medicines readily available on-site for patients[22]. By integrating the WHO/ISH prediction charts into the national guidelines for management of hypertension and CVD prevention in Bangladesh, the proportion of facilities using established guidelines may increase as the charts are easy to implement, interpret, and access. Additionally, since only one-third of facilities have essential CVD medicines readily available,

For peer review only

distributing pharmacologic treatment to those at highest risk of premature mortality due to CVD will be crucial.

The World Health Organization has outlined global targets in the Global Monitoring Framework for the control of NCDs in LMICs, which prioritizes an 80% of availability of affordable basic technologies and essential medicines necessary to treat significant NCDs, including CVDs in both rural and urban areas of the country. Additionally, the Framework recommends LMICs should target that at least 50% of eligible adults with a 10-year CVD risk of ≥30% receive drug therapy counseling to prevent heart attacks and strokes[4]. As such, limited CVD treatment options and weak health care infrastructure to access preventive services in Bangladesh is a significant public health concern. As public financing for health care is limited in Bangladesh (~1% of gross domestic product or GDP), cost-effective public health policies from national bodies on CVD drug treatment guidelines based on cost estimates and out of pocket costs to the population of Bangladesh is necessary for effective CVD control. Effective policies should address the potential for overtreatment, which comes at a high cost to both the health care system and the patient. The high percentage of the Bangladeshi adult population at low 10-year CVD risk (<10%) highlights the potential for reduction of CVD risk through population-wide public health policy and availability of accessible preventive services.

Limitations of this analytic approach should be considered when interpreting our results. CVD 10-year risk was developed using data compiled from each of the 14 WHO epidemiological sub-regions rather than individual countries. As such, the estimation used from each region's chart will most likely apply to the largest country within each region, or from the country where most of the data originated. However, over the past decade, the accuracy and predictive value for the current risk prediction charts have been evaluated and improved as more epidemiological data became available from individual countries[6 15]. Additionally, the charts provide approximate estimates of CVD risk in people who do not have established coronary heart disease, stroke or other atherosclerotic diseases. We were unable to confirm the medical

For peer review only - http://bmjopen.bmj.com/site/about/guidelines.xhtml

history of participants using medical charts or health records and relied on self-report, leading to the potential for measurement error and recall bias.

Additionally, we were unable to measure total cholesterol. Future research studies should include the measurement of total cholesterol using blood samples. Finally, our cost estimates were based on the prevalence of each risk approach in our study sample. Although we present the total number of people estimated to require drug treatment using 2016 census data, we were unable to identify population estimates of only those at risk of their first CVD event due to lack of surveillance data. Nevertheless, our data are still valuable as we focus on the comparative cost difference of each approach to underscore the potential cost savings in implementing the total risk approach in Bangladesh.

Conclusion

Our data show that the implementation of a total risk approach compared with a single risk factor approach will reduce the health care expenditure by lowering drug costs, which accounts for 60% of out-of-pocket spending in Bangladesh. This approach would be particularly beneficial in Bangladesh, a low-resource country that should prioritize the development of health policy for effective resource allocation in the public health sector. Furthermore, using the total risk approach would increase service coverage and allow for the distribution of resources to target those at highest risk of experiencing a heart attack or stroke. As the majority of the Bangladeshi adult population aged ≥40 years have a low ten-year risk of CVD, strategies that target those at highest risk of CVD coupled with public health policies to reduce the population-level risk of CVD may be effective.

For peer review only - http://bmjopen.bmj.com/site/about/guidelines.xhtml

Figure

Figure 1: Comparison of estimated annual costs using three different approaches to pharmacologic intervention for cardiovascular disease treatment among adults in Bangladesh aged 40 years and above

For peer review only

For peer review only - http://bmjopen.bmj.com/site/about/guidelines.xhtml

Abbreviations

WHO: World Health Organization

aPR: Adjusted prevalence ratio

BBS: Bangladesh Bureau of Statistics

BMI: Body mass index

BP: Blood pressure

CI: Confidence interval

CVD: Cardiovascular disease

ISH: International Society of Hypertension

NCD: Non-communicable disease

SEAR: South East-Asian Region

SBP: Systolic blood pressure

DBP: Diastolic blood pressure

PSU: Primary sampling unit

SD: Standard deviation

For peer review only

For peer review only - http://bmjopen.bmj.com/site/about/guidelines.xhtml

Footnotes

Ethics approval and consent to participate: We obtained ethical approval for this study from the Institutional Review Board of the National Institute of Ophthalmology (Study ID: 128). We provided participants with detailed study information using a printed handout prepared in Bengali to inform them of the objective of the study. Written consent was obtained from participants through signature or, if not possible, through thumbprint.

Competing Interests: The authors of this study declare no conflict of interest.

Funding: Technical and financial assistance for this study was provided by the WHO Country Office for Bangladesh.

Consent to publish: All authors consent to the publication of this manuscript.

Competing Interests: The authors declare no competing interests. The authors alone are responsible for views expressed in this article and they do not necessarily represent the views, decisions or policies of the institutions with which they are affiliated.

Availability of data and materials: The de-identified participant data used and/or analyzed during the current study are available from the corresponding author on reasonable request. Please contact M. Mostafa Zaman at zamanm@who.int for further information and guidelines.

Authors contributions: JYI: conceptualized the manuscript, analyzed data, interpreted results critically, and drafted the manuscript. MMZ: conceptualized the manuscript, designed the study, interpreted results critically, guided manuscript writing, and critically reviewed it.: trained the field team, implemented the survey, processed and analyzed data, and reviewed the manuscript.

Acknowledgments: The study was completed by the National Institute of Ophthalmology (NIO) of Bangladesh with technical assistance from the WHO Country Office for Bangladesh.

For peer review only - http://bmjopen.bmj.com/site/about/guidelines.xhtml

503 **References**

504 1. Checkley W, Ghannem H, Irazola V, et al. Management of NCD in low- and middle-income
505 countries. *Global heart* 2014;9(4):431-43. doi: 10.1016/j.gheart.2014.11.003

506 2. Islam SM, Purnat TD, Phuong NT, et al. Non-communicable diseases (NCDs) in developing
507 countries: a symposium report. *Globalization and health* 2014;10:81. doi:
508 10.1186/s12992-014-0081-9

509 3. Global action plan for the prevention and control of noncommunicable diseases 2013-2020.
510 Geneva, Switzerland: World Health Organization; 2013 [Available from:
511 http://apps.who.int/iris/bitstream/10665/94384/1/9789241506236_eng.pdf?ua=1
512 accessed 01/10 2019.

513 4. NCD Global Monitoring Framework: World Health Organization; 2017 [Available from:
514 http://www.who.int/nmh/global_monitoring_framework/en/ accessed 09/29 2017.

515 5. Ndindjock R, Gedeon J, Mendis S, et al. Potential impact of single-risk-factor versus total risk
516 management for the prevention of cardiovascular events in Seychelles. *Bulletin of the*
517 *World Health Organization* 2011;89(4):286-95. doi: 10.2471/BLT.10.082370

518 6. Mendis S, Lindholm LH, Anderson SG, et al. Total cardiovascular risk approach to improve
519 efficiency of cardiovascular prevention in resource constrain settings. *Journal of clinical*
520 *epidemiology* 2011;64(12):1451-62. doi: 10.1016/j.jclinepi.2011.02.001

521 7. Fatema K, Zwar NA, Milton AH, et al. Cardiovascular risk assessment among rural
522 population: findings from a cohort study in a peripheral region of Bangladesh. *Public*
523 *health* 2016;137:73-80. doi: 10.1016/j.puhe.2016.02.016

For peer review only - http://bmjopen.bmj.com/site/about/guidelines.xhtml

8. Fatema K, Zwar NA, Milton AH, et al. Application of two versions of the WHO/international society of hypertension absolute cardiovascular risk assessment tools in a rural Bangladeshi population. *BMJ open* 2015;5(10):e008140. doi: 10.1136/bmjopen-2015-008140

9. Fatema K, Rahman B, Zwar NA, et al. Short-term predictive ability of selected cardiovascular risk prediction models in a rural Bangladeshi population: a case-cohort study. *BMC cardiovascular disorders* 2016;16(1):105. doi: 10.1186/s12872-016-0279-2

10. Zaman MM, Rahman MM, Rahman MR, et al. Prevalence of risk factors for non-communicable diseases in Bangladesh: Results from STEPS survey 2010. *Indian journal of public health* 2016;60(1):17-25. doi: 10.4103/0019-557X.177290

11. Rahman M, Zaman MM, Islam JY, et al. Prevalence, treatment patterns, and risk factors of hypertension and pre-hypertension among Bangladeshi adults. *Journal of human hypertension* 2018;32(5):334-48. doi: 10.1038/s41371-017-0018-x

12. Karim MN, Zaman MM, Rahman MM, et al. Sociodemographic Determinants of Low Fruit and Vegetable Consumption Among Bangladeshi Adults: Results From WHO-STEPS Survey 2010. *Asia-Pacific journal of public health / Asia-Pacific Academic Consortium for Public Health* 2017;29(3):189-98. doi: 10.1177/1010539517699059

13. Population and Housing Census 2001, updated in 2009 Dhaka, Bangladesh: Bangladesh Bureau of Statistics 2009 [Available from: http://microdata.worldbank.org/index.php/catalog/1616 accessed 01/10 2019.

14. Global report on diabetes Geneva, Switzerland: World Health Organization 2016 [Available from: https://www.who.int/diabetes/global-report/en/.

For peer review only

For peer review only - http://bmjopen.bmj.com/site/about/guidelines.xhtml

15. Mendis S, Lindholm LH, Mancia G, et al. World Health Organization (WHO) and International Society of Hypertension (ISH) risk prediction charts: assessment of cardiovascular risk for prevention and control of cardiovascular disease in low and middle-income countries. *Journal of hypertension* 2007;25(8):1578-82. doi: 10.1097/HJH.0b013e3282861fd3

16. Population Projection of Bangladesh Dynamics and Trends 2011-2061 Dhaka, Bangladesh Bangladesh Bureau of Statistics 2015 [Available from: http://203.112.218.65:8008/WebTestApplication/userfiles/Image/PopMonographs/PopulationProjection.pdf accessed 11/17 2018.

17. Cravedi P, Sharma SK, Bravo RF, et al. Preventing renal and cardiovascular risk by renal function assessment: insights from a cross-sectional study in low-income countries and the USA. *BMJ open* 2012;2(5) doi: 10.1136/bmjopen-2012-001357

18. Multisectoral Action Plan for the Noncommunicable Disease Control and Prevention (2016-2021) Dhaka, Bangladesh: Ministry of Health and Family Welfare, Government of the People's Republic Bangladesh.; 2015 [Available from: http://www.searo.who.int/entity/ncd_tobacco_surveillance/monitoring_fw/pages_from_ban_ncd_action_plan_2016_2021_me_not_approved.pdf accessed 12/15 2018.

19. Non Communicable Diseases in Bangladesh: Current Scenario and Future Directions Dhaka, Bangladesh2017 [Available from: http://www.brac.net/images/news/2017/bhw_report2016_final.pdf accessed 05/10 2019.

20. Health and Morbidity Status Survey-2012 Dhaka, Bangladesh: Bangladesh Bureau of Statistics, Ministry of Planning.; 2013 [Available from:

For peer review only - http://bmjopen.bmj.com/site/about/guidelines.xhtml

http://bbs.portal.gov.bd/sites/default/files/files/bbs.portal.gov.bd/page/4c7eb0f0_e780_4686_b546_b4fa0a8889a5/HMSS.pdf accessed 12/25 2018.

21. Noncommunicable Diseases Progress Monitor 2017 Geneva, Switzerland: World Health Organization; 2017 [231]. Available from: https://www.who.int/nmh/publications/ncd-progress-monitor-2017/en/ accessed 02/15 2019.

22. NIPORT. Bangladesh Health Facility Survey 2014 Dhaka, Bangladesh: National Institute of Population Research and Training (NIPORT), Associates for Community and Population Research (ACPR), and ICF International. ; 2016 [Available from: https://dhsprogram.com/pubs/pdf/SPA23/SPA23.pdf accessed 03/15 2019.

For peer review only - http://bmjopen.bmj.com/site/about/guidelines.xhtml

Table 1: Background Characteristics of Bangladeshi Adult Participants (n = 6189)

	Total (n = 6189)			Men (n = 2824)			Women (n = 3365)		
Characteristics	Mean (SD)	n	%	Mean (SD)	n	%	Mean (SD)	n	%
Age (years)	52.9 (9.9)			53.5 (10.1)			52.5 (9.8)		
Education (years)	3.1 (4.2)			4.3 (4.8)			2.1 (3.3)		
Area of Residence									
Urban		1873	30.2		808	28.6		1065	31.6
Rural		4316	69.8		2016	71.4		2300	68.4
Occupation									
Professional employment[‡]		1007	16.3		880	31.3		127	3.8
Industrial worker/Day Laborer		1600	25.9		1440	51.2		160	4.8
Housework		2697	43.7		-	-		2697	80.3
Unemployed/Retired		735	11.9		386	13.7		349	10.4
Other[§]		131	2.1		106	3.8		25	0.7
Smoking Tobacco Use[‖]									
Current User		1493	24.2		1432	50.7		61	1.8
Ever User		2261	36.5		2150	76.1		111	3.3
Smokeless Tobacco Use[◊]									
Current User		3078	49.7		1208	42.8		1870	55.6
Ever User		3469	56.1		1433	50.7		2046	60.5
Alcohol use in the last 30 days									
Yes		75	1.2		68	2.4		7	0.2
Body Mass Index[¶]	21.9 (4.1)			21.4 (3.7)			22.3 (4.4)		
Waist Cirumference[**] (cm)	82.4 (13.3)			81.8 (10.5)			82.8 (15.2)		
Blood Pressure									
Systolic Blood Pressure[††](mmHg)	119.7 (15.2)			119.8 (14.9)			119.7 (15.5)		
Diastolic Blood Pressure[‡‡] (mmHg)	80.3 (9.5)			80.2 (9.4)			80.3 (9.7)		
Blood Glucose Levels[§§] (mmol/L)	6.9 (3.0)			6.9 (3.0)			6.9 (2.9)		

Abbreviations: SD: Standard Deviation; CM: centimeters; mmHg: millimeter of mercury; mmol/L: millimoles per liter

[‡]Professional occupation includes: Government employee, private company employee, businessman

[§]Other occupation includes: Shop keeper, weavers, driver, student, beggar, cook, carpenter, and tailor

For peer review only

For peer review only - http://bmjopen.bmj.com/site/about/guidelines.xhtml

||Excluding smokeless tobacco | Missing values, n = 18; Men, n = 11; FeMen, n = 7

◊ Smokeless tobacco use includes jorda, white leaf (shaada pata), gul, etc.

¶Body mass index (BMI) calculated by weight in kilogram divided by height in meter squared

For peer review only

For peer review only - http://bmjopen.bmj.com/site/about/guidelines.xhtml

Table 2: Ten-year risk of combined myocardial infarction and stroke (fatal and non-fatal) by gender, using the WHO/ISH SEAR-D charts without cholesterol (n = 6090)

	Total (n = 6090)			Men (n = 2779)			Women (n = 3311)			*P**
	n	%	CI	n	%	CI	n	%	CI	
Low Risk (<10 %)	5189	85.2	84.3 - 86.1	2309	83.1	81.6 - 84.4	2880	87.0	85.8 - 88.1	<0.001
Moderate Risk (10 - <20%)	760	12.5	11.6 - 13.3	391	14.1	12.8 - 15.4	369	11.1	10.1 - 12.2	0.001
High Risk (20 - <30%)	107	1.8	1.4 - 2.1	60	2.2	1.7 - 2.8	47	1.4	1.0 - 1.9	0.029
Very High Risk (≥ 30%)	34	0.6	0.4 - 0.8	19	0.7	0.4 - 1.1	15	0.5	0.3 - 0.7	0.229

Abbreviations: CI, confidence interval; WHO, World Health Organization; ISH, International Society of Hypertension; SEAR, South East Asian Region
* P-value based on chi-square test

For peer review only

For peer review only - http://bmjopen.bmj.com/site/about/guidelines.xhtml

Table 3: Estimation of percentage of population requiring drug treatment based on total risk approach in comparison to single risk factor approaches (n = 6090)

	Total Risk Approach				Single Risk Factor Approach
	Cardiovascular Risk ≥ 30%		Cardiovascular Risk ≥ 20%		
	CV Risk ≥ 30% alone, %	CV Risk ≥ 30% + BP ≥ 160/100	CV Risk ≥ 20% alone, %	CV Risk ≥ 20% + BP ≥ 160/100	BP ≥ 140/90 (SBP ≥ 140 + isolated raised DBP), %
Men	0.68	4.3	2.8	3.5	18.8
omen	0.45	4.8	1.9	4.1	20.2
Total	0.56	4.5	2.3	3.8	19.6

Abbreviations: CV, cardiovascular; BP, blood pressure; SBP, systolic blood pressure; DBP, diastolic blood pressure

For peer review only

For peer review only - http://bmjopen.bmj.com/site/about/guidelines.xhtml

Table 4: Population projections and comparison of medicine costs (US$) for implementing total risk approach vs. single risk factor approach in Bangladesh SEAR-D

Total risk approach CV Risk ≥ 30% alone

	Total no. of people in the general population (in thousands)*	Percentage of population aged ≥40 years requiring medication (%)	Total no. of people estimated to require drug treatment (in thousands)	No. of people per million population (aged 40 years and older)	Estimated annual total cost of CVD medication treatment per million population† Aspirin	Enalapril	Hydrochlorothiazide	Simvastatine	Total
Age Group									
Men									
40 to 49	9210	0.1	921	1000	$1,642.5	$8,687.0	$3,029.5	$15,549.0	$28,908.0
50 to 59	6303	0.4	2521	4000	$6,570.0	$34,748.0	$12,118.0	$62,196.0	$115,632.0
60 to 69	3730	2.5	9325	25000	$41,062.5	$217,175.0	$75,737.5	$388,725.0	$722,700.0
>= 70	1881	0.7	1317	7000	$11,497.5	$60,809.0	$21,206.5	$108,843.0	$202,356.0
Total	21124	0.7	14787	7000	$11,497.5	$60,809.0	$21,206.5	$108,843.0	$202,356.0
Women									
40 to 49	9087	0.0	0	0	$0.0	$0.0	$0.0	$0.0	$0.0
50 to 59	5662	0.3	1699	3000	$4,927.5	$26,061.0	$9,088.5	$46,647.0	$86,724.0
60 to 69	3257	0.4	1303	4000	$6,570.0	$34,748.0	$12,118.0	$62,196.0	$115,632.0
>= 70	1638	3.3	5405	33000	$54,202.5	$286,671.0	$99,973.5	$513,117.0	$953,964.0
Total	19644	0.5	9822	5000	$8,212.5	$43,435.0	$15,147.5	$77,745.0	$144,540.0
All									
40 to 49	18296	0.0	732	400	$657.0	$3,474.8	$1,211.8	$6,219.6	$11,563.2
50 to 59	11965	0.3	3590	3000	$4,927.5	$26,061.0	$9,088.5	$46,647.0	$86,724.0
60 to 69	6989	1.3	9086	13000	$21,352.5	$112,931.0	$39,383.5	$202,137.0	$375,804.0

For peer review only - http://bmjopen.bmj.com/site/about/guidelines.xhtml

>= 70	3518	2.0	7036	20000	$32,850.0	$173,740.0	$60,590.0	$310,980.0	$578,160.0
Total	40768	0.6	24461	6000	$9,855.0	$52,122.0	$18,177.0	$93,294.0	$173,448.0

Total risk approach CV Risk ≥ 20% alone

	Total no. of people in the general population (in thousands)*	Percentage of population aged ≥40 years requiring medication (%)	Total no. of people estimated to require drug treatment (in thousands)	No. of people per million population (aged 40 years and older)	Aspirin	Enalapril	Hydrochlorothiazide	Simvastatine	Total
Age Group									
Men									
40 to 49	9210	1.9	17499	19000	$31,207.5	$165,053.0	$57,560.5	$295,431.0	$549,252.0
50 to 59	6303	1.6	10085	16000	$26,280.0	$138,992.0	$48,472.0	$248,784.0	$462,528.0
60 to 69	3730	4.1	15293	41000	$67,342.5	$356,167.0	$124,209.5	$637,509.0	$1,185,228.0
>= 70	1881	2.4	4514	24000	$39,420.0	$208,488.0	$72,708.0	$373,176.0	$693,792.0
Total	21124	2.3	48585	23000	$37,777.5	$199,801.0	$69,678.5	$357,627.0	$664,884.0
Women									
40 to 49	9087	0.8	7270	8000	$13,140.0	$69,496.0	$24,236.0	$124,392.0	$231,264.0
50 to 59	5662	2.4	13589	24000	$39,420.0	$208,488.0	$72,708.0	$373,176.0	$693,792.0
60 to 69	3257	2.0	6514	20000	$32,850.0	$173,740.0	$60,590.0	$310,980.0	$578,160.0
>= 70	1638	7.4	12121	74000	$121,545.0	$642,838.0	$224,183.0	$1,150,626.0	$2,139,192.0
Total	19644	2.1	41252	21000	$34,492.5	$182,427.0	$63,619.5	$326,529.0	$607,068.0
All									
40 to 49	18296	1.3	23785	13000	$21,352.5	$112,931.0	$39,383.5	$202,137.0	$375,804.0
50 to 59	11965	2.0	23930	20000	$32,850.0	$173,740.0	$60,590.0	$310,980.0	$578,160.0
60 to 69	6989	2.9	20268	29000	$47,632.5	$251,923.0	$87,855.5	$450,921.0	$838,332.0
>= 70	3518	4.9	17238	49000	$80,482.5	$425,663.0	$148,445.5	$761,901.0	$1,416,492.0
Total	40768	2.2	89690	22000	$36,135.0	$191,114.0	$66,649.0	$342,078.0	$635,976.0

Single Risk Factor Approach: BP ≥ 140/90 (SBP ≥ 140 + isolated raised DBP), %

For peer review only - http://bmjopen.bmj.com/site/about/guidelines.xhtml

For peer review only

	Total no. of people in the general population (in thousands)*	Percentage of population aged ≥40 years requiring medication (%)	Total no. of people estimated to require drug treatment (in thousands)	No. of people per million population (aged 40 years and older)	Aspirin	Enalapril	Hydrochlorothiazide	Simvastatine	Total
Age Group									
Men									
40 to 49	9210	14.5	133545	145000	$238,162.5	$1,259,615.0	$439,277.5	$2,254,605.0	$4,191,660.0
50 to 59	6303	18.4	115975	184000	$302,220.0	$1,598,408.0	$557,428.0	$2,861,016.0	$5,319,072.0
60 to 69	3730	24.7	92131	247000	$405,697.5	$2,145,689.0	$748,286.5	$3,840,603.0	$7,140,276.0
>= 70	1881	26.0	48906	260000	$427,050.0	$2,258,620.0	$787,670.0	$4,042,740.0	$7,516,080.0
Total	21124	18.8	397131	188000	$308,790.0	$1,633,156.0	$569,546.0	$2,923,212.0	$5,434,704.0
Women									
40 to 49	9087	16.9	153570	169000	$277,582.5	$1,468,103.0	$511,985.5	$2,627,781.0	$4,885,452.0
50 to 59	5662	23.8	134756	238000	$390,915.0	$2,067,506.0	$721,021.0	$3,700,662.0	$6,880,104.0
60 to 69	3257	21.3	69374	213000	$349,852.5	$1,850,331.0	$645,283.5	$3,311,937.0	$6,157,404.0
>= 70	1638	23.5	38493	235000	$385,987.5	$2,041,445.0	$711,932.5	$3,654,015.0	$6,793,380.0
Total	19644	20.2	396809	202000	$331,785.0	$1,754,774.0	$611,959.0	$3,140,898.0	$5,839,416.0
All									
40 to 49	18296	15.8	289077	158000	$259,515.0	$1,372,546.0	$478,661.0	$2,456,742.0	$4,567,464.0
50 to 59	11965	21.2	253658	212000	$348,210.0	$1,841,644.0	$642,254.0	$3,296,388.0	$6,128,496.0
60 to 69	6989	22.8	159349	228000	$374,490.0	$1,980,636.0	$690,726.0	$3,545,172.0	$6,591,024.0
>= 70	3518	24.7	86895	247000	$405,697.5	$2,145,689.0	$748,286.5	$3,840,603.0	$7,140,276.0
Total	40768	19.6	799053	196000	$321,930.0	$1,702,652.0	$593,782.0	$3,047,604.0	$5,665,968.0

*Source: 2016 Population Projections (using 2011 Census data) Bangladesh Bureau of Statistics, Statistics and Informatics Division, Ministry of Planning

† Price for 100 tablets in US$: Aspirin (0.45), Enalapril (2.38), Hydrochlorothiazide (0.83), Simvastatine (4.29); one tablet is taken per day

For peer review only

For peer review only - http://bmjopen.bmj.com/site/about/guidelines.xhtml

Appendix Table 1: Cost of common drugs used to treat cardiovascular disease in Bangladesh (converted to US$)

Drug Name	Dose	Number of Tablets	Category	Median price (in Bangladeshi Taka)	Price (in US$)*
Aspirin	100 mg	100 tablets	Antiplatelet drugs	38BDT	0.45
Atenolol	50 mg	100 tablets	Antihypertensive, Beta-adrenoreceptor blocking drugs	77BDT	0.92
Captopril	25 mg	100 tablets	Antihypertensive, ACE inhibitors	300 BDT	3.58
Chrlorthalidone	25 mg	30 tablets	Antihypertensive, thiazide diuretics	60 BDT	0.72
Enalapril	10 mg	100 tablets	Antihypertensive, ACE inhibitors	200 BDT	2.38
Frusemide	40 mg	100 tablets	Antihypertensive, loop diuretics	53 BDT	0.63
Hydrochlorothiazide	25 mg	100 tablets	Antihypertensive, thiazide diuretics	70 BDT	0.83
Isosorbide dinitrate	10 mg	100 tablets	Angina treatment, nitrates - coronary vasodilators	35 BDT	0.42
Labetalol	100 mg	60 tablets	Antihypertensive, Beta-adrenoreceptor blocking drugs	361.2 BDT	4.31
Losartan	50 mg	100 tablets	Antihypertensive, Angiotensin-II receptor blocker	600 BDT	7.15
Lovastatine	20 mg	30 tablets	Lipid regulation, statin	303.3 BDT	3.62
Methyldopa	250 mg	100 tables	Antihypertensive, Centrally acting or central sympatholytic	308 BDT	3.67
Nifedipine (slow release)	20 mg	100 tablets	Angina treatment, calcium-channel blocker	64 BDT	0.76
Propranolol hydrochloride	10 mg	100 tablets	Antihypertensive, Beta-adrenoreceptor blocking drugs	24 BDT	0.29
Simvastatine	10 mg	30 tablets	Lipid regulation, statin	360 BDT	4.29

For peer review only

Spironolactone	25 mg	100 tablets	Antihypertensive, potassium-sparing diuretics & aldoesterone antagonist	202 BDT	2.41
Streptokinase	1.5 million unit/vial	One vial	Anticoagulant	3100 BDT	36.96
Warfarin	5 mg	100 tablets	Anticoagulant	300 BDT	3.58

Abbreviations: mg, milligrams; US, United States; BDT, Bangladeshi Taka

*Price conversion based on exchange rate on 02/07/2019

For peer review only - http://bmjopen.bmj.com/site/about/guidelines.xhtml

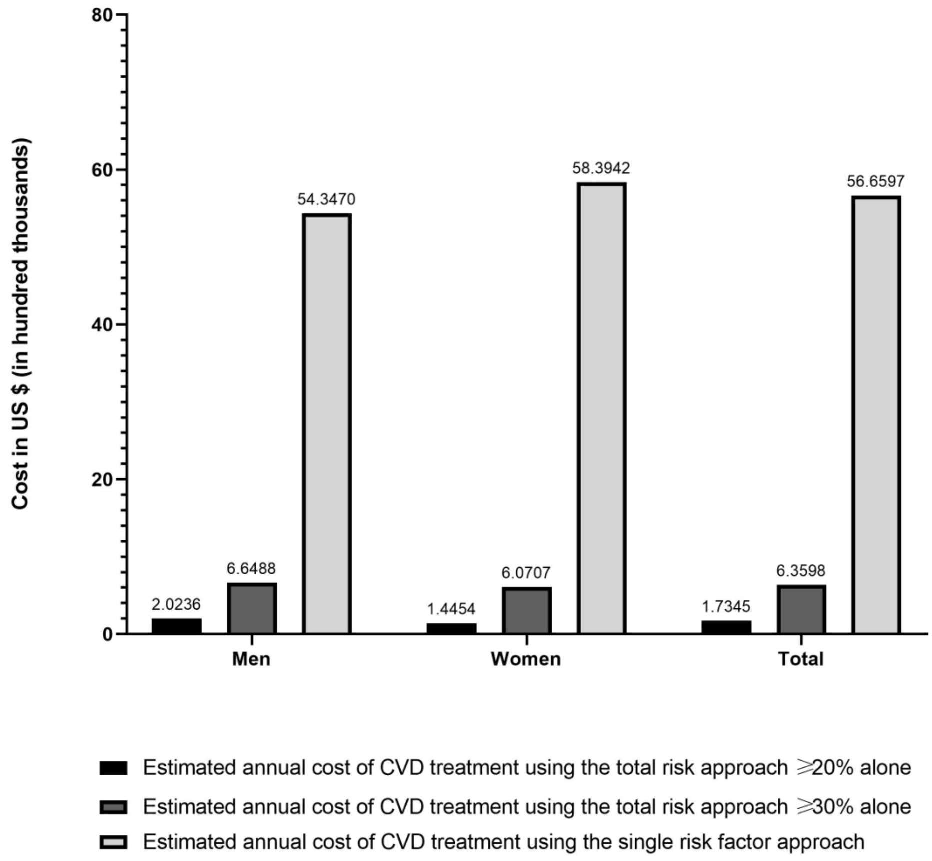

omparison of estimated annual costs using three different approaches to pharmacologic intervention for cardiovascular disease treatment among adults in Bangladesh aged 40 years and above

196x175mm (300 x 300 DPI)

STROBE 2007 (v4) Statement—Checklist of items that should be included in reports of *cross-sectional studies*

Section/Topic	Item #	Recommendation	Reported on page #
**Title and abstract**	1	(*a*) Indicate the study's design with a commonly used term in the title or the abstract	2
		(*b*) Provide in the abstract an informative and balanced summary of what was done and what was found	2-3
**Introduction**			
Background/rationale	2	Explain the scientific background and rationale for the investigation being reported	5-6
Objectives	3	State specific objectives, including any prespecified hypotheses	6
**Methods**			
Study design	4	Present key elements of study design early in the paper	6
Setting	5	Describe the setting, locations, and relevant dates, including periods of recruitment, exposure, follow-up, and data collection	7
Participants	6	(*a*) Give the eligibility criteria, and the sources and methods of selection of participants	6
Variables	7	Clearly define all outcomes, exposures, predictors, potential confounders, and effect modifiers. Give diagnostic criteria, if applicable	8-10
Data sources/ measurement	8*	For each variable of interest, give sources of data and details of methods of assessment (measurement). Describe comparability of assessment methods if there is more than one group	7-10
Bias	9	Describe any efforts to address potential sources of bias	6-7 & 15-16
Study size	10	Explain how the study size was arrived at	7
Quantitative variables	11	Explain how quantitative variables were handled in the analyses. If applicable, describe which groupings were chosen and why	9-10
Statistical methods	12	(*a*) Describe all statistical methods, including those used to control for confounding	9-10
		(*b*) Describe any methods used to examine subgroups and interactions	8-11
		(*c*) Explain how missing data were addressed	9
		(*d*) If applicable, describe analytical methods taking account of sampling strategy	N/A
		(*e*) Describe any sensitivity analyses	N/A
**Results**			

For peer review only

Participants	13*	(a) Report numbers of individuals at each stage of study—eg numbers potentially eligible, examined for eligibility, confirmed eligible, included in the study, completing follow-up, and analysed	10
		(b) Give reasons for non-participation at each stage	N/A
		(c) Consider use of a flow diagram	N/A
Descriptive data	14*	(a) Give characteristics of study participants (eg demographic, clinical, social) and information on exposures and potential confounders	10
		(b) Indicate number of participants with missing data for each variable of interest	9
Outcome data	15*	Report numbers of outcome events or summary measures	
Main results	16	(*a*) Give unadjusted estimates and, if applicable, confounder-adjusted estimates and their precision (eg, 95% confidence interval). Make clear which confounders were adjusted for and why they were included	N/A
		(*b*) Report category boundaries when continuous variables were categorized	N/A
		(*c*) If relevant, consider translating estimates of relative risk into absolute risk for a meaningful time period	N/A
Other analyses	17	Report other analyses done—eg analyses of subgroups and interactions, and sensitivity analyses	10
**Discussion**			
Key results	18	Summarise key results with reference to study objectives	12-14
Limitations	19	Discuss limitations of the study, taking into account sources of5potential bias or imprecision. Discuss both direction and magnitude of any potential bias	15-16
Interpretation	20	Give a cautious overall interpretation of results considering objectives, limitations, multiplicity of analyses, results from similar studies, and other relevant evidence	13-16
Generalisability	21	Discuss the generalisability (external validity) of the study results	15
**Other information**			
Funding	22	Give the source of funding and the role of the funders for the present study and, if applicable, for the original study on which the present article is based	19

*Give information separately for cases and controls in case-control studies and, if applicable, for exposed and unexposed groups in cohort and cross-sectional studies.

Note: An Explanation and Elaboration article discusses each checklist item and gives methodological background and published examples of transparent reporting. The STROBE checklist is best used in conjunction with this article (freely available on the Web sites of PLoS Medicine at http://www.plosmedicine.org/, Annals of Internal Medicine at http://www.annals.org/, and Epidemiology at http://www.epidem.com/). Information on the STROBE Initiative is available at www.strobe-statement.org.

For peer review only

BMJ Open

Estimation of total cardiovascular risk using the 2019 WHO CVD prediction charts and comparison of population-level costs based on alternative drug therapy guidelines in Bangladesh: a population-based study of adults in Bangladesh

Journal:	*BMJ Open*
Manuscript ID	bmjopen-2019-035842.R1
Article Type:	Original research
Date Submitted by the Author:	06-Feb-2020

Complete List of Authors:	Islam, Jessica; World Health Organization Country Office for Bangladesh, ; University of North Carolina at Chapel Hill Gillings School of Global Public Health, Department of Epidemiology<br>Zaman, MM; World Health Organization, Dhaka, Bangladesh,<br>Moniruzzaman, Mohammad; Shiga University of Medical Science, Public Health<br>Ara Shakoor, Shawkat; National Institute of Opthalmology<br>Hossain, A.H.M. Enayet; National Institute of Opthalmology

**Primary Subject Heading**:	Epidemiology
Secondary Subject Heading:	Cardiovascular medicine
Keywords:	Cardiac Epidemiology < CARDIOLOGY, EPIDEMIOLOGY, Hypertension < CARDIOLOGY, Ischaemic heart disease < CARDIOLOGY, Myocardial infarction < CARDIOLOGY

SCHOLARONE™
Manuscripts

I, the Submitting Author has the right to grant and does grant on behalf of all authors of the Work (as defined in the below author licence), an exclusive licence and/or a non-exclusive licence for contributions from authors who are: i) UK Crown employees; ii) where BMJ has agreed a CC-BY licence shall apply, and/or iii) in accordance with the terms applicable for US Federal Government officers or employees acting as part of their official duties; on a worldwide, perpetual, irrevocable, royalty-free basis to BMJ Publishing Group Ltd ("BMJ") its licensees and where the relevant Journal is co-owned by BMJ to the co-owners of the Journal, to publish the Work in this journal and any other BMJ products and to exploit all rights, as set out in our licence.

The Submitting Author accepts and understands that any supply made under these terms is made by BMJ to the Submitting Author unless you are acting as an employee on behalf of your employer or a postgraduate student of an affiliated institution which is paying any applicable article publishing charge ("APC") for Open Access articles. Where the Submitting Author wishes to make the Work available on an Open Access basis (and intends to pay the relevant APC), the terms of reuse of such Open Access shall be governed by a Creative Commons licence – details of these licences and which Creative Commons licence will apply to this Work are set out in our licence referred to above.

Other than as permitted in any relevant BMJ Author's Self Archiving Policies, I confirm this Work has not been accepted for publication elsewhere, is not being considered for publication elsewhere and does not duplicate material already published. I confirm all authors consent to publication of this Work and authorise the granting of this licence.

For peer review only

Estimation of total cardiovascular risk using the 2019 WHO CVD prediction charts and comparison of population-level costs based on alternative drug therapy guidelines in Bangladesh: a population-based study of adults in Bangladesh

Running title: Total cardiovascular risk approach in Bangladesh

Authors: Jessica Yasmine Islam[1], M Mostafa Zaman[2], Mohammad Moniruzzaman[3], Shawkat Ara Shakoor[4], A.H.M. Enayet Hossain[4]

Author Affiliations:

[1]Department of Epidemiology, Gillings School of Global Public Health, University of North Carolina at Chapel Hill, North Carolina, USA

[2]Research and Publication Unit, World Health Organization, Dhaka, Bangladesh

[3]Center for Epidemiologic Research in Asia, Shiga University of Medical Science, Otsu, Shiga, Japan

[4]National Institute of Ophthalmology. Sher-e-Bangla Nagar, Dhaka, Bangladesh

Corresponding Author:

Dr. Jessica Yasmine Islam, PhD, MPH
Department of Epidemiology
UNC Gillings School of Global Public Health
UNC Chapel Hill
135 Dauer Drive
Chapel Hill, NC 27599
Email: islamjy@email.unc.edu

For peer review only

Abstract

Objective: The objective of this study is to estimate the population distribution of 10-year cardiovascular disease (CVD) risk among Bangladeshi adults aged 40 years and above, using the 2019 WHO CVD prediction charts. Additionally, we compared the cost of pharmacologic treatment of CVD based on CVD risk (thresholds ≥ 30%/ ≥ 20%) to single risk factor (hypertension) cutoff levels in the Bangladeshi context.

Study Design: Cross-sectional, population-based study

Setting and Participants: From 2013-214, we collected data from a nationally representative cross-sectional survey of adults aged ≥40 years from urban and rural areas of Bangladesh (n = 6189). Using the 2019 World Health Organization risk prediction charts, CVD risk was calculated and categorized as very low (<5%), low (5-<10%), moderate (10-<20%), high (20-<30%), and very high risk (≥30%). We estimated drug therapy costs using the lowest price of each drug class available (aspirin, thiazide diuretics, statins, and angiotensin-converting enzyme inhibitors). We compared the total cost of drug therapy using the total risk vs. single risk factor approach.

Primary Outcome Measures: Our primary outcome was 10-year CVD risk categorized as very low (<5%), low (5-<10%), moderate (10-<20%), high (20-<30%), and very high risk (≥30%).

Results: The majority of adults (85.2%, 95% CI: 84.3–86.1) have a 10-year risk of CVD of less than 10%. The proportion of adults with a 10-year CVD risk of ≥20% was 0.51%. Only one adult was categorized with a 10-year CVD risk of ≥30%. Among adults with CVD risk groups of very low, low, and moderate, 17.4%, 27.9%, and 41.4% had hypertension (BP ≥140/90) and 0.1%, 1.7%, and 2.9% had severe

For peer review only - http://bmjopen.bmj.com/site/about/guidelines.xhtml

hypertension (BP≥160/100), respectively. Using the total risk approach would reduce drug costs per million populations to $144,540 (risk of ≥ 20%).

Conclusion: To reduce health care expenditure for the prevention and treatment of CVD, a total risk approach using the WHO/ISH risk prediction chart may lead to cost-savings.

Keywords: non-communicable diseases, Bangladesh, cardiovascular disease, pharmacologic intervention, cost, chronic disease

For peer review only

Article Summary

Strengths and Limitations of the Study

- Using the recently updated 2019 WHO CVD Risk Prediction Charts, this study provides evidence for incorporating the WHO/ISH CVD Risk Prediction Charts into CVD management and health care guidelines, and may lead to potential cost-savings from a societal perspective.
- The 2019 WHO/ISH CVD Risk Prediction Charts should be applied to a population who have not experienced a CVD event in the past, however, we were unable to confirm self-reported medical history of participants using medical charts or health records, leading to the potential for measurement error due to recall bias.
- The cost estimates we present are an underestimate of total costs for CVD-related treatment as the focus of this study is on cost of pharmacologic intervention only as the largest contributor to overall direct costs in Bangladesh.
- Although we present the total number of people estimated to require drug treatment using 2014 population data, we were unable to identify population estimates of only those at risk of their first CVD event due to lack of surveillance data.
- The CVD 10-year risk cut-offs were defined using risk prediction models derived from 85 cohorts mostly from high-income countries, as data from large-scale prospective cohort data from most low-income and middle-income countries (LMICs) are unavailable.

For peer review only

Background

Globally, cardiovascular disease (CVD) is the leading cause of death and disproportionately impacts low- and middle-income countries (LMICs), where over 75% of CVD-related deaths occur (1). People living in LMICs are at high risk of developing CVDs due to the absence of integrated primary care for early detection and prevention of CVD-related risk factors, such as high cholesterol, high blood pressure, and smoking. Limited access to primary care and the growing burden of CVDs is a significant cause of poverty in LMICs and hinders the macroeconomic development of many countries(2). LMICs are estimated to experience cumulative economic losses exceeding 7 trillion US dollars over the next 15 years due to morbidity and mortality caused by noncommunicable diseases, including CVD(3). As such, the significance of the CVD epidemic has gained increasing international recognition over the past decade, leading to the development of several international guidelines for CVD control and prevention(4).

In 2007, the World Health Organization (WHO) published pocket guidelines, including CVD risk prediction charts, designed for health care workers in LMICs to guide patient 10-year risk stratification for heart attack or stroke(5). There are two possible strategies suitable for a low resource setting to assess the risk of a cardiovascular event and identify those at high risk of a fatal CVD outcome: 1. Utilize a single risk factor management strategy, which focuses on one condition at a time, such as hypertension; or, 2. Utilize a more holistic approach considering several risk factors such as age, tobacco use, gender, diabetes diagnosis, body mass index, blood pressure, and blood cholesterol when measured. Through the total risk approach, pocket guidelines help to identify high-risk patients that are in imminent danger of a heart attack or stroke for timely pharmacologic treatment or surgical interventions. Additionally, applying the total risk approach via the WHO prediction charts in a nationally representative sample provides an opportunity to estimate and monitor population-level distribution of CVD risk to ultimately inform CVD treatment policy recommendations (6, 7).

Currently, Bangladesh has not incorporated clinical guidelines for screening or treatment of risk factors based on absolute CVD risk scores and estimation of the population distribution of CVD risk over time. Data are needed to support the implementation of the WHO/ISH prediction charts as clinical

For peer review only - http://bmjopen.bmj.com/site/about/guidelines.xhtml

guidelines for Bangladesh, a resource-limited setting, to demonstrate the potential cost-savings and benefit of the WHO recommendation on CVD prevention. Recently in 2019, the World Health Organization updated the CVD risk charts based on newly validated risk prediction models to estimate CVD risk in 21 Global Burden of Disease regions(8). The newly developed risk predictions models have been calibrated using data from The Global Burden of Disease study to include estimates from low- and middle-income countries. To our knowledge, here we present the first analysis to apply the updated CVD risk charts among a cohort of Bangladeshi adults. Prior studies conducted in Bangladesh have estimated CVD risk among adults residing in rural areas only and have not included a nationally representative population (9-11). Additionally, no prior studies have estimated the potential costs of pharmacological treatment for CVD in Bangladesh using either the single risk factor or total risk approach, as done previously in other settings(7). As such, our objective was to assess the distribution of absolute CVD risk among a nationally representative sample of Bangladeshi adults using the 2019 WHO risk prediction chart recommended for the WHO South Asian Region (Bangladesh, Bhutan, India, Nepal, and Pakistan) We also compared the costs of drug treatments for CVD prevention using the total cardiovascular risk thresholds at ≥20% and with single risk factor cutoff levels (blood pressure ≥ 140/90 mm Hg).

For peer review only

Methods

Study design and setting

Data were analyzed from a population-based cross-sectional study conducted from September to December 2013 to assess the burden of blindness and low vision among adults in Bangladesh. The target population of this survey included men and women residing in Bangladesh over the age of 40 years. The exclusion criteria included tourists and the institutionalized, such as residents of a military base, hospital, prisons, nursing homes, and other such institutions. We obtained ethical approval for this study from the Institutional Review Board of the National Institute of Ophthalmology. We provided participants with detailed information regarding the study objectives and procedures using a printed handout prepared in

For peer review only - http://bmjopen.bmj.com/site/about/guidelines.xhtml

Bengali. Written consent was obtained from participants through signature or, if not possible, through thumbprint.

Sampling Frame

We adopted a multistage, geographically clustered, probability-based sampling approach to obtain a nationally representative sample of Bangladesh, as previously described and outlined per the WHO STEPwise approach (12-15). Population statistics were obtained using the 2011 national census conducted by the Bangladesh Bureau of Statistics (BBS) to create the sample frame (16). The sampling frame included 64,407 primary sampling units (PSUs) covering all seven divisions of the country. We randomly selected seventy-two PSUs (25 urban and 47 rural) from seven divisions, with the probability of selection proportional to the population size of each division. In each PSU, we selected 100 consecutive households as the secondary sampling unit.

For each household, a trained field data collector approached the head of the household or the family member most knowledgeable of the residents to screen for eligible participants. The screening respondent was asked to describe the composition of household residents, which was defined as those who considered the home to be their primary place of residence as of the night before. A list was composed and ordered from youngest to oldest age in years starting from 40 years. Using the list of eligible residents, we used the Kish table approach to randomly select one participant from each home. The selected participant was asked to come to a nearby health center the next day to administer the survey by trained study interviewers and undergo a medical examination by the study physician. Based on the medical review, participants were followed-up with by the providers at the health center for treatment.

Patient and Public Involvement

There was no patient or public involvement in the implementation of this study or interpretation of analytic results.

For peer review only - http://bmjopen.bmj.com/site/about/guidelines.xhtml

Data Collection

To ensure effective and uniform data collection, field interviewers underwent a seven-day training on the interview methodology by the study ophthalmologists and epidemiologists. The training included an in-depth review of the survey content and protocol for completing the demographic questionnaire (a modified WHO/PBL Version III). Each member of the data collection team was provided a detailed survey protocol manual outlining the survey activities, the questionnaire interview, and information about the duties and responsibilities of all survey personnel.

Demographic data were collected, including age, sex, marital status, educational level, and occupation, using a structured questionnaire survey. Data regarding tobacco use, health history, and treatment history were also collected. Participants were asked if they smoked (e.g. cigarette, hookah, pipe) or if they used smokeless tobacco (e.g. chewing tobacco, jorda) to assess history of tobacco use. Each participant provided medical history for a prior diagnosis of high blood pressure or hypertension, diabetes, renal disease, or any cardiovascular diseases by a health care provider. Medication history was obtained including medication for high blood pressure, diabetes, malaria, steroids, tuberculosis, and among women, history of oral contraception. The questionnaire was translated from English to Bengali, adapted, and validated before data collection.

Physical measurements, including height, weight, and blood pressure (BP) were collected. Trained field interviewers measured BP using an appropriately calibrated aneroid sphygmomanometer with appropriately sized arm cuffs. BP measurements were consistently taken on the right arm at heart level and elbow-assisted while the participant was seated. The initial measurement was performed after five minutes of rest. After two minutes, the second measurement was taken. The mean of these two BP readings was utilized as the final BP for each participant. To measure blood glucose levels, we obtained random blood glucose samples (17). Capillary blood samples were consistently taken using the right arm and index finger with a glucometer (Accu-chek Advantage, Roche Diagnostics Division, Grenzacherstrasse, Switzerland).

For peer review only - http://bmjopen.bmj.com/site/about/guidelines.xhtml

Estimation of 10-year CVD risk

We estimated 10-year CVD risk using the 2019 WHO CVD risk prediction charts(8, 18). The prediction charts provide the 10-year risk of a fatal or non-fatal major cardiovascular event, such as myocardial infarction or stroke, based on age, sex, blood pressure, body mass index, smoking status, total blood cholesterol, and the presence or absence of diabetes mellitus for 14 WHO epidemiological sub-regions. For each region, two sets of charts have been developed based on the availability of laboratory-based results. As total cholesterol was not measured in our cohort, we utilized the WHO cardiovascular disease risk non-laboratory-based charts developed for South Asia (including, Bangladesh, Bhutan, India, Nepal, and Pakistan). The non-laboratory-based risk charts do not account for diabetes diagnosis or total cholesterol level.

The prediction chart grades cardiovascular risk using the following categories: age (1: 40–44years; 2: 45-49 years; 3: 50-54 years; 4: 55-59 years; 5: 60-64 years; 6: 64-69 years; 7: 70-74 years), sex (men and women), smoking (smoker or non-smoker), systolic blood pressure (<120 mmHg, 120 to 139, 140 to 159, 160 - <180, and ≥180), and body mass index (<20, 20-24, 25-29, 30-35, and ≥35). The risk categories for 10-year combined acute myocardial infarction and stroke (fatal and non-fatal) are as follows: <5%, 5-<10%, 10 - <20%, 20 to <30%, and ≥ 30%.

Observations with missing values were dropped from the analysis. We did not anticipate any bias from the complete-case analysis approach as the number of missing observations for key variables was less than 2%: smoking status, n=18 or 0.3% missing values; systolic blood pressure, n = 22 or 0.4% missing values; diastolic blood pressure, n = 25 or 0.4% missing values; and body mass index, n=30 or 0.5%,

Data Analysis

We present sociodemographic variables using mean (standard deviation) or the median (interquartile range) for continuous variables and proportion for categorical variables. We conducted bivariate analyses by sex and age group. We used the chi-square test to assess for any significant

For peer review only - http://bmjopen.bmj.com/site/about/guidelines.xhtml

differences in CVD risk distribution across sex. For estimating the cost of medicines per million per year (population aged 40 years or older), we used the lowest price of each drug class available in the market (generic preparation of aspirin, thiazide diuretics, statins, and angiotensin-converting enzyme inhibitors). Appendix 1 includes further details regarding the specific costs of common drugs used to treat cardiovascular disease in Bangladesh.

T0 calculate costs we included the following categories: (1) people with high cardiovascular risk (≥20% and BP ≥160/100), who are recommended for pharmacological intervention using four different types of drugs for treatment(5, 7); and (2) people with BP ≥140/90, who are recommended antihypertensive treatment. To calculate the estimated annual total cost of CVD medication treatment per million populations (aged 40 years or older), we multiplied the percentage of the population at risk and the price of medicine in Bangladesh. We included an estimate of the total number of people estimated to require drug treatment as follows: we multiplied the prevalence of the population requiring medication based on each approach based on our study estimates, by the number of people in the general population in 2013 (19) stratified by gender and age group.

Results

Demographic Characteristics

The mean age of included participants was 52.9 years (men: 53.5 years, women: 52.5 years) (Table 1). The average level of educational attainment was 3.1 years of education; women were generally less educated than men (2.1 years vs. 4.3 years, respectively). The majority (80%) of women were housewives, and among men, the most common occupation was an industrial worker or day laborer (51.2%). Overall, over one-third of participants ever used smoking tobacco, and over half ever used smokeless tobacco. Few participants drank alcohol in the past 30 days (1.2%). The mean BMI was 21.9 kg/m^{2}, and the mean waist circumference was 82.4 cm.

Overall, the prevalence of hypertension (defined as BP ≥140/90) increased with age among men and women. Additionally, women had higher prevalence of hypertension among nearly all age-groups in

For peer review only - http://bmjopen.bmj.com/site/about/guidelines.xhtml

both urban and rural areas. Prevalence of hyperglycemia (glucose ≥11.1 mmol/L) was higher among urban adults compared to rural across all age groups. The highest prevalence of hyperglycemia was observed among urban men aged 60-69 years at 18.2%. Finally, the prevalence of overweight and obesity was higher among urban residents than rural residents. The largest prevalence of overweight and obesity were observed among urban women, with prevalence as high as 51.4% among women aged 40-49 years (Figure 1).

Distribution of Cardiovascular Risk

We summarized the distribution of cardiovascular risk in the population overall and stratified by sex in Table 2. Eighty-five percent of participants had a low (< 10%) 10-year cardiovascular risk, and this proportion was significantly different across sex (p<0.001). Over half (63.7%) of women had a very low (<5%) cardiovascular risk. Almost all (99.5%) of the study population were categorized as having cardiovascular risk < 20%. A higher proportion of men (1.0%) were categorized as high risk than women (0.1%) (p = <0.001). Overall, only one male participant was categorized as very high risk or with a cardiovascular risk of ≥ 30%.

We summarized the prevalence of adults with hypertension by CVD risk group (Figure 2). Among those with 10-<20% CVD risk, we observed a high proportion of hypertensive (41.4%). In the high risk group (≥20%), 100% had hypertension. Additionally, among those with ≥20% CVD risk, we observed that 35% had severe hypertension (BP ≥160/100).

Cost of Drug Treatment

We were unable to compare costs of drug treatment at two cardiovascular risk thresholds (30% to 20%) due to only one male adult with a CVD risk at ≥30%. We observed low proportion of adults with CVD risk ≥20% at 0.5%. When we included BP ≥160/100 measurements, the number of people requiring treatment more than tripled from 0.5% to 1.8% (Table 3). Conversely, if a single risk factor approach was applied, and all those with hypertension (a persistent SBP ≥140 and/or DBP ≥90) were treated,24.6%of

For peer review only - http://bmjopen.bmj.com/site/about/guidelines.xhtml

the sample would require drug treatment, specifically antihypertensive; more than 20 times the proportion identified when using the total cardiovascular risk approach alone.

Comparison of Cost by Approach

Next, we compared the estimated annual cost of medicines per million populations for implementing the total risk approach vs. the single risk factor approach. Table 4 shows the estimated number of people aged 40 years or older requiring drug treatment stratified by age group and gender. The estimates showed that if the single risk factor approach is applied in Bangladesh with its percentage of population at risk and the lowest price of medicine in the country, the cost per million populations (aged 40 years or older) of treating those with BP ≥140/90 would be $7,111,368 US$; if the absolute risk approach were applied, the cost of treating those with 10-year risk of CVD ≥20% per million populations (aged 40 years or older) would be $144,540, almost fifty times less (Figure 3). The cost estimation was based on the percentage of the population at different levels of risk and the differences in the price of generic medicines. For this analysis, we focused on the cost of pharmacological treatment as it is the most critical contributor to the overall direct costs of CVD treatment in Bangladesh. We assumed that other costs of CVD treatment and prevention service delivery, such as health facilities and wages of health workers, are similar for both approaches of service delivery(7).

Discussion

Using this nationally representative survey of Bangladeshi adults aged 40 years and above, we found that the majority of adults (97%) were at a very low, low or moderate ten-year risk of myocardial infarction and stroke. The proportion of adults requiring drug treatment rose from 1.0% to 2.1% when the threshold for pharmacologic intervention was changed from≥20% only to ≥20% and blood pressure of 160/100, respectively; which was lower the proportion than the single risk factor approach (24.6%). Our data demonstrated that using a single risk factor approach to manage individual cardiovascular risk factors is costlier ($7,111,368per million population) than using the total risk approach (CVD risk ≥20, $144,540

For peer review only - http://bmjopen.bmj.com/site/about/guidelines.xhtml

per million population), as a more substantial proportion of adults will need drug treatment. Findings from this analysis support the implementation of clinical guidelines using CVD risk scores calculated using the WHO/ISH prediction charts to appropriately identify patients at highest risk of CVD development over ten years in Bangladesh.

In our study using nationally representative data, we found that the 10-year risk of CVD was low (<10%) among the vast majority of adults (85.1%). Additionally, only 0.5% of adults were at high risk (≥ 20%) of a CVD event within the next ten years. In this analysis, we applied the 2019 CVD Risk Charts for South Asia, which are newly developed and now incorporate body mass index as part of the prediction chart algorithm. Our results are comparable to data from South Asia presented in the 2019 Lancet publication by the WHO CVD Risk Chart Working Group, which showed that 0% of women from both Bhutan and Nepal had a CVD risk level above 20%. Similarly, 0% of men from Bhutan, and only <2% of men from Nepal were categorized with a risk level above 20%(8). These data demonstrate a lower prevalence of CVD risk ≥20% than prior reports from South Asia, which utilized the original risk prediction charts published in 2007. For example, prior data from Nepal showed that 4.3% of adults were categorized with a high (≥20%) 10-year risk of a CVD event(20). Further, analyses from a rural area of south India revealed that seventeen percent of participants had moderate to high risk (10->20%) of cardiovascular events per the 2007 WHO prediction charts(21). Finally, data collected in 2010 from Pakistan showed that 10% of adults were categorized with ≥20% CVD risk, with 2.9% as high as ≥40%(7). When utilizing the 2019 WHO prediction charts on the population level to measure and monitor trends in total CVD risk in recent years, policy makers should interpret the trends with caution, and potentially compare changes in trends of CVD-risk using the criteria of both the 2007 and 2019 WHO risk prediction charts.

Our data demonstrate a similar drop in proportion of adults with a CVD risk ≥20% as observed in other South Asian countries when the 2019 risk prediction charts are applied. Utilizing the 2007 prediction charts, two prior studies have reported absolute CVD risk among the Bangladeshi population (10, 22). When using the prior version of the 2007 WHO risk prediction charts Fatema et al. found that

For peer review only - http://bmjopen.bmj.com/site/about/guidelines.xhtml

10% of Bangladeshi adults living in rural areas were at high risk (≥ 20%) of a CVD event within the next ten years, and half of these adults fell in the very high-risk category (≥ 30%). These proportions were the same when total cholesterol was incorporated in the WHO/ISH CVD Risk-Assessment Tool. Unfortunately, we were unable to measure total blood cholesterol and were unable to make similar comparisons. In another rural Bangladeshi population, the proportion of participants at a high-risk (≥ 20%) of a CVD event in 10 years was 2.1%, which is closer in proportion to our finding(22). No other studies in Bangladesh have been conducted to assess the 10-year risk of CVD using the WHO/ISH tool. We present novel data using the 2019 WHO risk prediction charts and a nationally representative sample, which may be generalizable to the population of Bangladesh. Data we present may be used to inform policymakers decisions on clinical guidelines and resource allocation for treatment of CVDs in Bangladesh.

Similar to prior analyses conducted using data from eight low- and middle-income countries(7), our results demonstrate that in the Bangladeshi context using a single risk factor approach to evaluate risk of CVD-related mortality would cost more than implementing the total risk approach due to higher drug costs. In Bangladesh, about 60% of out-of-pocket expenditure patients face goes towards drugs directly bought from pharmacies, diagnostics, and informal providers(23). Additionally, patients in Bangladesh experience a high level of uncertainty regarding out-of-pocket costs due to unforeseen costs on diagnostics and medications, including under-the-table payments to providers for reliable and timely services(24). Currently, Bangladesh does not offer universal health coverage or affordable health insurance plans. The cost of treatment for CVD frequently leads to catastrophic health expenditure and impoverishment; the proportion of catastrophic spending for treatment is highest among those from the lowest quintiles of wealth (14%) compared to those with high wealth and high socioeconomic status (6.6%) (25). As such, implementing the WHO risk prediction charts may be beneficial to patients in Bangladesh as only those at highest risk of future CVD would be recommended for life-saving treatment.

In addition to benefits to the patient, implementing the total risk approach would also be beneficial to the health care system in Bangladesh by improving NCD preventive service delivery and the

For peer review only - http://bmjopen.bmj.com/site/about/guidelines.xhtml

use of guidelines for adequate care. Currently, Bangladesh is categorized by the World Bank as a lower-middle-income country with emerging health challenges as the burden of NCDs continues to grow. In 2015, an estimated 67% of all deaths in Bangladesh were due to NCDs and the risk of premature death from chronic disease among adults aged 30-70 years was 22%(26). Indeed, CVDs and circulatory diseases are the leading cause of mortality and morbidity in Bangladesh. Despite this substantial burden, preventive services for CVDs in Bangladesh are limited. In 2014, an estimated 16% of health care facilities across the country (i.e. hospitals, community clinics) had the resources to diagnose, prescribe treatment for, and manage patients with CVDs(27). District hospitals (95%) and upazila health complexes (81%), and private hospitals (77%) were more likely to provide services for cardiovascular diseases than other facilities. Only 10% of community clinics and maternal and child welfare centers, and 17% of union level facilities, which are the most accessible providers in rural areas, provided any cardiovascular services, and the services at these facilities were limited to the measurement of blood pressure or referrals(27). Among facilities with the capacity to offer services for CVD management, about only 20% utilized established guidelines for hypertension treatment and less than one-third had essential CVD medicines readily available on-site for patients(27). By integrating the WHO risk prediction charts into the national guidelines for management of hypertension and CVD prevention in Bangladesh, the proportion of facilities using established guidelines may increase as the charts are easy to implement, interpret, and access. Additionally, since only one-third of facilities have essential CVD medicines readily available, distributing pharmacologic treatment to those at highest risk of premature mortality due to CVD will be crucial.

Although implementation of a total risk approach may lead to cost-savings, the WHO prediction charts may underestimate CVD risk in certain categories of people such as those with persistent raised blood pressure ≥160/100 mmHg, blood cholesterol ≥8 mmol/L, or those suffering from diabetes with renal disease(5). Patients who may fall in these categories should be recommended for intensive lifestyle interventions, and appropriate drug therapy. In fact, the risk models used to develop the 2019 CVD risk charts may have underestimated CVD risk due to limitations in the population data used to estimate

For peer review only - http://bmjopen.bmj.com/site/about/guidelines.xhtml

incidences: As reported in the 2019 Lancet publication by the WHO CVD Risk Chart Working Group the data used to develop the predictions models likely included people already on cardiovascular disease prevention therapies, such as statins, which have led to an underestimate in CVD risk(8). In our study, we underscore the potential for underestimation of CVD risk by comparing the proportion of adults categorized as high risk (≥20% CVD risk) to those who would be diagnosed with hypertension (BP ≥ 140/60) and severe hypertension (BP ≥ 160/100). Additionally, we provided a graphical summary of common risk factors of CVD, including hypertension, hyperglycemia, and overweight and obesity. Despite our very low proportion of adults who would be recommended for treatment based on the risk prediction charts, we observed a high prevalence of these risk factors particularly in urban populations.

Limitations of this analytic approach should be considered when interpreting our results. The CVD 10-year risk cut-offs were defined using risk prediction models derived from 85 cohorts mostly from high-income countries, as data from large-scale prospective cohort data from most low-income and middle-income countries (LMICs) are unavailable. Data were used from the Global Burden of Disease project to recalibrate the models to be representative of LMICs, however, the GBD data do not have country-specific disease risk estimates. As such, the estimation used from each region's chart will most likely apply to the largest country within each region, or from the country where most of the data originated. The risk prediction he charts provide approximate estimates of CVD risk in people who do not have established coronary heart disease, stroke or other atherosclerotic diseases. Although we included simvastatin in our pharmacologic cost analysis, we were unable to measure total cholesterol or confirm the medical history of participants using medical charts and relied on self-report, leading to the potential for measurement error and recall bias. Further, our data were collected in 2013 and may be outdated as population growth in older age groups has been observed in recent years. Our analyses should be replicated using more recent data and future research studies should include the measurement of total cholesterol. Finally, our cost estimates were based on the prevalence of each risk approach in our study sample. Although we present the total number of people estimated to require drug treatment using 2013 population data, estimates of only those at risk of their first CVD event were unavailable due to lack of

For peer review only - http://bmjopen.bmj.com/site/about/guidelines.xhtml

surveillance data. Nevertheless, our data are valuable as the first analysis to apply the 2019 WHO CVD risk prediction charts to a cohort of adults in Bangladesh. Additionally, we provide data on the comparative cost difference of each approach to underscore the potential cost savings in implementing the total risk approach in Bangladesh. Cost data presented in this analysis may be used in future cost-effectiveness analyses to compare the total risk and single risk factor approach when considering all costs from a societal perspective to inform health policy in Bangladesh.

The World Health Organization has outlined global targets in the Global Monitoring Framework for the control of NCDs in LMICs, which prioritizes an 80% of availability of affordable basic technologies and essential medicines necessary to treat significant NCDs, including CVDs in both rural and urban areas of the country. Limited CVD treatment access and weak health care infrastructure in Bangladesh is a significant public health concern. As public financing for health care is limited in Bangladesh (~1% of gross domestic product or GDP), public health policies on CVD drug treatment guidelines based on cost estimates, such as out-of-pocket costs is necessary for effective CVD control. Effective policies should address the potential for overtreatment, which comes at a high cost to both the health care system and the patient. The high percentage of the Bangladeshi adult population at low 10-year CVD risk (<10%) highlights the potential for reduction of CVD risk through population-wide public health policy and availability of accessible preventive services. However, caution should be taken to ensure that risk stratification approaches are not used in inappropriate clinical circumstances, such as adults with highly uncontrolled hypertension with blood pressure measurements at 160/100 mmHg.

Conclusion

Our data show that the implementation of a total risk approach compared with a single risk factor approach will reduce the health care expenditure by lowering drug costs, which accounts for 60% of out-of-pocket spending in Bangladesh. This approach would be particularly beneficial in Bangladesh, a low-resource country that should prioritize the development of health policy for effective resource allocation in the public health sector. Using the total risk approach would increase service coverage and allow for

For peer review only - http://bmjopen.bmj.com/site/about/guidelines.xhtml

the distribution of resources to target those at highest risk of experiencing a heart attack or stroke. As the majority of the Bangladeshi adult population aged ≥40 years have a low ten-year risk of CVD, strategies that target those at highest risk of CVD coupled with public health policies to reduce the population-level risk of CVD may be effective.

For peer review only

For peer review only - http://bmjopen.bmj.com/site/about/guidelines.xhtml

Footnotes

Acknowledgments: The study was completed by the National Institute of Ophthalmology (NIO) of Bangladesh with technical assistance from the WHO Country Office for Bangladesh. The authors are grateful to the management and field team of NIO consisting of ophthalmologists, other doctors, nurses, technologists, and enumerators for their hard works.

Competing Interests: The authors of this study declare no conflict of interest. The authors alone are responsible for the views expressed in this article and they do not necessarily represent the views, decisions or policies of the institutions with which they are affiliated.

Funding: Financial assistance for this study was provided by the WHO Country Office for Bangladesh (WHO Reference: 2013/355662-0, Purchase Order: 200843353, Reg. File: BAN-2013-B7-TSA-0001)

Consent to publish: All authors consent to the publication of this manuscript.

Availability of data and materials: The de-identified participant data used and/or analyzed during the current study are available from the corresponding author on reasonable request. Please contact M. Mostafa Zaman at zamanm@who.int for further information and guidelines.

Authors contributions: JYI: conceptualized the manuscript, analyzed data, interpreted results critically, and drafted the manuscript. MMZ: conceptualized the manuscript, designed the study, interpreted results critically, guided manuscript writing, and critically reviewed it. MM, SAS, AHMEH prepared the survey protocol, trained the field team, implemented the survey, processed the data, and reviewed the manuscript. SAS is the guarantor of data.

For peer review only

Figures:

Figure 1: Prevalence of hypertension, hyperglycemia, and overweight and obesity among Bangladeshi adults aged 40 years and above by area of residence and age group

Figure 2: Proportion of adults with hypertension or severe hypertension by CVD risk group

Figure 3: Annual costs of pharmacologic treatment for CVD by sex and risk stratification approach

For peer review only

References

1. Checkley W, Ghannem H, Irazola V, Kimaiyo S, Levitt NS, Miranda JJ, et al. Management of NCD in low- and middle-income countries. Global heart. 2014;9(4):431-43. doi: 10.1016/j.gheart.2014.11.003. PubMed PMID: 25592798; PubMed Central PMCID: PMCPMC4299752.

2. Islam SM, Purnat TD, Phuong NT, Mwingira U, Schacht K, Froschl G. Non-communicable diseases (NCDs) in developing countries: a symposium report. Globalization and health. 2014;10:81. doi: 10.1186/s12992-014-0081-9. PubMed PMID: 25498459; PubMed Central PMCID: PMCPMC4267750.

3. Global action plan for the prevention and control of noncommunicable diseases 2013-2020. Geneva, Switzerland: World Health Organization; 2013 [cited 2019 01/10]. Available from: http://apps.who.int/iris/bitstream/10665/94384/1/9789241506236_eng.pdf?ua=1.

4. NCD Global Monitoring Framework: World Health Organization; 2017 [cited 2017 09/29]. Available from: http://www.who.int/nmh/global_monitoring_framework/en/.

5. Prevention of cardiovascular disease: pocket guidelines for assessment and management of cardiovascular disease Geneva: World Health Organization, 2007.

6. Ndindjock R, Gedeon J, Mendis S, Paccaud F, Bovet P. Potential impact of single-risk-factor versus total risk management for the prevention of cardiovascular events in Seychelles. Bulletin of the World Health Organization. 2011;89(4):286-95. doi: 10.2471/BLT.10.082370. PubMed PMID: 21479093; PubMed Central PMCID: PMCPMC3066521.

7. Mendis S, Lindholm LH, Anderson SG, Alwan A, Koju R, Onwubere BJ, et al. Total cardiovascular risk approach to improve efficiency of cardiovascular prevention in resource constrain settings. Journal of clinical epidemiology. 2011;64(12):1451-62. doi: 10.1016/j.jclinepi.2011.02.001. PubMed PMID: 21530172.

For peer review only - http://bmjopen.bmj.com/site/about/guidelines.xhtml

8. Group WCRCW. World Health Organization cardiovascular disease risk charts: revised models to estimate risk in 21 global regions. The Lancet Global health. 2019;7(10):e1332-e45. doi: 10.1016/S2214-109X(19)30318-3. PubMed PMID: 31488387.

9. Fatema K, Zwar NA, Milton AH, Rahman B, Awal AS, Ali L. Cardiovascular risk assessment among rural population: findings from a cohort study in a peripheral region of Bangladesh. Public health. 2016;137:73-80. doi: 10.1016/j.puhe.2016.02.016. PubMed PMID: 27036981.

10. Fatema K, Zwar NA, Milton AH, Rahman B, Ali L. Application of two versions of the WHO/international society of hypertension absolute cardiovascular risk assessment tools in a rural Bangladeshi population. BMJ open. 2015;5(10):e008140. doi: 10.1136/bmjopen-2015-008140. PubMed PMID: 26463220; PubMed Central PMCID: PMCPMC4606419.

11. Fatema K, Rahman B, Zwar NA, Milton AH, Ali L. Short-term predictive ability of selected cardiovascular risk prediction models in a rural Bangladeshi population: a case-cohort study. BMC cardiovascular disorders. 2016;16(1):105. doi: 10.1186/s12872-016-0279-2. PubMed PMID: 27386836; PubMed Central PMCID: PMCPMC4937534.

12. Zaman MM, Rahman MM, Rahman MR, Bhuiyan MR, Karim MN, Chowdhury MA. Prevalence of risk factors for non-communicable diseases in Bangladesh: Results from STEPS survey 2010. Indian journal of public health. 2016;60(1):17-25. doi: 10.4103/0019-557X.177290. PubMed PMID: 26911213; PubMed Central PMCID: PMCPMC6349138.

13. Rahman M, Zaman MM, Islam JY, Chowdhury J, Ahsan HN, Rahman R, et al. Prevalence, treatment patterns, and risk factors of hypertension and pre-hypertension among Bangladeshi adults. Journal of human hypertension. 2018;32(5):334-48. doi: 10.1038/s41371-017-0018-x. PubMed PMID: 29230005; PubMed Central PMCID: PMCPMC5860750.

For peer review only

14. Karim MN, Zaman MM, Rahman MM, Chowdhury MAJ, Ahsan H, Hassan MM, et al. Sociodemographic Determinants of Low Fruit and Vegetable Consumption Among Bangladeshi Adults: Results From WHO-STEPS Survey 2010. Asia-Pacific journal of public health / Asia-Pacific Academic Consortium for Public Health. 2017;29(3):189-98. doi: 10.1177/1010539517699059. PubMed PMID: 28434246; PubMed Central PMCID: PMCPMC6349139.

15. Surveillance of risk factors for noncommunicable disease: the WHO STEPwise approach. Geneva: World Health Organization, 2001.

16. Population and Housing Census 2001, updated in 2009 Dhaka, Bangladesh: Bangladesh Bureau of Statistics 2009 [cited 2019 01/10]. Available from: http://microdata.worldbank.org/index.php/catalog/1616.

17. Global report on diabetes Geneva, Switzerland: World Health Organization 2016. Available from: https://www.who.int/diabetes/global-report/en/.

18. Mendis S, Lindholm LH, Mancia G, Whitworth J, Alderman M, Lim S, et al. World Health Organization (WHO) and International Society of Hypertension (ISH) risk prediction charts: assessment of cardiovascular risk for prevention and control of cardiovascular disease in low and middle-income countries. Journal of hypertension. 2007;25(8):1578-82. doi: 10.1097/HJH.0b013e3282861fd3. PubMed PMID: 17620952.

19. Population Projection of Bangladesh Dynamics and Trends 2011-2061 Dhaka, Bangladesh Bangladesh Bureau of Statistics 2015 [cited 2018 11/17]. Available from: http://203.112.218.65:8008/WebTestApplication/userfiles/Image/PopMonographs/PopulationProjection.pdf.

20. Khanal MK, Ahmed MS, Moniruzzaman M, Banik PC, Dhungana RR, Bhandari P, et al. Total cardiovascular risk for next 10 years among rural population of Nepal using WHO/ISH risk prediction

For peer review only

For peer review only - http://bmjopen.bmj.com/site/about/guidelines.xhtml

chart. BMC research notes. 2017;10(1):120. doi: 10.1186/s13104-017-2436-9. PubMed PMID: 28270186; PubMed Central PMCID: PMCPMC5341399.

21. Ghorpade AG, Shrivastava SR, Kar SS, Sarkar S, Majgi SM, Roy G. Estimation of the cardiovascular risk using World Health Organization/International Society of Hypertension (WHO/ISH) risk prediction charts in a rural population of South India. Int J Health Policy Manag. 2015;4(8):531-6. doi: 10.15171/ijhpm.2015.88. PubMed PMID: 26340393; PubMed Central PMCID: PMCPMC4529043.

22. Cravedi P, Sharma SK, Bravo RF, Islam N, Tchokhonelidze I, Ghimire M, et al. Preventing renal and cardiovascular risk by renal function assessment: insights from a cross-sectional study in low-income countries and the USA. BMJ open. 2012;2(5). doi: 10.1136/bmjopen-2012-001357. PubMed PMID: 23002161; PubMed Central PMCID: PMCPMC3467605.

23. Multisectoral Action Plan for the Noncommunicable Disease Control and Prevention (2016-2021) Dhaka, Bangladesh: Ministry of Health and Family Welfare, Government of the People's Republic Bangladesh.; 2015 [cited 2018 12/15]. Available from: http://www.searo.who.int/entity/ncd_tobacco_surveillance/monitoring_fw/pages_from_ban_ncd_action_plan_2016_2021_me_not_approved.pdf.

24. Non Communicable Diseases in Bangladesh: Current Scenario and Future Directions Dhaka, Bangladesh2017 [cited 2019 05/10]. Available from: http://www.brac.net/images/news/2017/bhw_report2016_final.pdf.

25. Health and Morbidity Status Survey-2012 Dhaka, Bangladesh: Bangladesh Bureau of Statistics, Ministry of Planning.; 2013 [cited 2018 12/25]. Available from: http://bbs.portal.gov.bd/sites/default/files/files/bbs.portal.gov.bd/page/4c7eb0f0_e780_4686_b546_b4fa0a8889a5/HMSS.pdf.

For peer review only - http://bmjopen.bmj.com/site/about/guidelines.xhtml

26. Noncommunicable Diseases Progress Monitor 2017 Geneva, Switzerland: World Health Organization; 2017 [cited 2019 02/15]. 231]. Available from: https://www.who.int/nmh/publications/ncd-progress-monitor-2017/en/.

27. NIPORT. Bangladesh Health Facility Survey 2014 Dhaka, Bangladesh: National Institute of Population Research and Training (NIPORT), Associates for Community and Population Research (ACPR), and ICF International. ; 2016 [cited 2019 03/15]. Available from: https://dhsprogram.com/pubs/pdf/SPA23/SPA23.pdf.

For peer review only

For peer review only - http://bmjopen.bmj.com/site/about/guidelines.xhtml

Table 1: Background Characteristics of Bangladeshi Adult Participants (n = 6189)

	Total (n = 6189)			Men (n = 2824)			Women (n = 3365)		
Characteristics	Mean (SD)	n	%	Mean (SD)	n	%	Mean (SD)	n	%
Age (years)	52.9 (9.9)			53.5 (10.1)			52.5 (9.8)		
Education (years)	3.1 (4.2)			4.3 (4.8)			2.1 (3.3)		
Area of Residence									
Urban		1873	30.2		808	28.6		1065	31.6
Rural		4316	69.8		2016	71.4		2300	68.4
Occupation									
Professional employment[‡]		1007	16.3		880	31.3		127	3.8
Industrial worker/Day Laborer		1600	25.9		1440	51.2		160	4.8
Housework		2697	43.7		-	-		2697	80.3
Unemployed/Retired		735	11.9		386	13.7		349	10.4
Other[§]		131	2.1		106	3.8		25	0.7
Smoking Tobacco Use[ǁ]									
Current User		1493	24.2		1432	50.7		61	1.8
Ever User		2261	36.5		2150	76.1		111	3.3
Smokeless Tobacco Use[◊]									
Current User		3078	49.7		1208	42.8		1870	55.6
Ever User		3469	56.1		1433	50.7		2046	60.5
Alcohol use in the last 30 days									
Yes		75	1.2		68	2.4		7	0.2
Body Mass Index[¶]	21.9 (4.1)			21.4 (3.7)			22.3 (4.4)		
Waist Circumference[**] (cm)	82.4 (13.3)			81.8 (10.5)			82.8 (15.2)		
Blood Pressure									
Systolic Blood Pressure[††](mmHg)	119.7 (15.2)			119.8 (14.9)			119.7 (15.5)		
Diastolic Blood Pressure[‡‡] (mmHg)	80.3 (9.5)			80.2 (9.4)			80.3 (9.7)		
Blood Glucose Levels[§§] (mmol/L)	6.9 (3.0)			6.9 (3.0)			6.9 (2.9)		

Abbreviations: SD: Standard Deviation; CM: centimeters; mmHg: millimeter of mercury; mmol/L: millimoles per liter

[‡]Professional occupation includes: Government employee, private company employee, businessman

[§]Other occupation includes: Shop keeper, weavers, driver, student, beggar, cook, carpenter, and tailor

[ǁ]Excluding smokeless tobacco | Missing values, n = 18; Men, n = 11; Women, n = 7

[◊] Smokeless tobacco use includes jorda, white leaf (shaada pata), gul, etc.

[¶]Body mass index (BMI) calculated by weight in kilogram divided by height in meter squared

For peer review only - http://bmjopen.bmj.com/site/about/guidelines.xhtml

Table 2: Ten-year risk of combined myocardial infarction and stroke (fatal and non-fatal) by gender, using the 2019 WHO CVD Risk non-laboratory based charts for South Asia (n = 5977)

	Total (n = 5977)			Men (n = 2708)			Women (n = 3269)			*P**
	n	%	CI	n	%	CI	n	%	CI	
Very Low Risk (<5%)	3115	52.1	50.8-53.4	1034	38.2	36.6-40.0	2081	63.7	62.0-65.3	<0.001
Low Risk (5-10 %)	1972	33.0	31.8-34.2	1047	38.7	36.8-40.5	925	28.3	26.8-30.0	<0.001
Moderate Risk (10 - <20%)	860	14.4	13.5-15.3	600	22.2	20.6-23.8	260	7.8	7.0-8.9	<0.001
High Risk (20 - <30%)	29	0.5	0.3-0.7	26	1.0	0.6-1.4	3	0.1	0.0-0.2	<0.001
Very High Risk (≥ 30%)	1	0.0	0.0-0.01	1	0.0	0.0-0.1	0	0.0	0.0-0.0	0.272

Abbreviations: CI, confidence interval; WHO, World Health Organization; CVD, Cardiovascular disease

* P-value based on chi-square test

For peer review only

Table 3: Estimation of percentage of population requiring drug treatment based on total risk approach in comparison to single risk factor approaches (n = 5977)

	Cardiovascular Risk ≥ 20%		Single Risk Factor Approach
	CV Risk ≥ 20% alone, %	CV Risk ≥ 20% + BP ≥ 160/100	BP ≥ 140/90 (SBP ≥ 140 + isolated raised DBP), %
Men	1.0	2.1	22.1
Women	0.1	1.6	26.7
Total	0.5	1.8	24.6

Abbreviations: CV, cardiovascular; BP, blood pressure; SBP, systolic blood pressure; DBP, diastolic blood pressure

For peer review only

For peer review only - http://bmjopen.bmj.com/site/about/guidelines.xhtml

Table 4: Population projections and comparison of medicine costs (US$) for implementing total risk approach vs. single risk factor approach in Bangladesh

Total risk approach CV Risk ≥ 20% alone

	Total no. of people in the general population (in thousands)*	Percentage of population aged ≥40 years requiring medication (%)	Total no. of people estimated to require drug treatment (in thousands)	No. of people per million population (aged 40 years and older)	Estimated annual total cost of CVD medication treatment per million population† Aspirin	Enalapril	Hydrochlorothiazide	Simvastatin	Total
Age Group									
Men									
40 to 49	9210	0.4	3660	4000	$6,570.0	$34,748.0	$12,118.0	$62,196.0	$115,632.0
50 to 59	6303	0.1	596	1000	$1,642.5	$8,687.0	$3,029.5	$15,549.0	$28,908.0
60 to 69	3730	1.5	4733	15000	$24,637.5	$130,305.0	$45,442.5	$233,235.0	$433,620.0
>= 70	1881	7.0	17507	70000	$114,975.0	$608,090.0	$212,065.0	$1,088,430.0	$2,023,560.0
Total	21124	1.0	20769	10000	$16,425.0	$86,870.0	$30,295.0	$155,490.0	$289,080.0
Women									
40 to 49	9087	0.0	0	0	$0.0	$0.0	$0.0	$0.0	$0.0
50 to 59	5662	0.0	0	0	$0.0	$0.0	$0.0	$0.0	$0.0
60 to 69	3257	0.0	0	0	$0.0	$0.0	$0.0	$0.0	$0.0
>= 70	1638	1.6	3965	16000	$26,280.0	$138,992.0	$48,472.0	$248,784.0	$462,528.0
Total	19644	0.1	1958	1000	$1,642.5	$8,687.0	$3,029.5	$15,549.0	$28,908.0
All									
40 to 49	18296	0.2	3605	2000	$3,285.0	$17,374.0	$6,059.0	$31,098.0	$57,816.0
50 to 59	11965	0.1	1134	1000	$1,642.5	$8,687.0	$3,029.5	$15,549.0	$28,908.0
60 to 69	6989	0.7	4206	7000	$11,497.5	$60,809.0	$21,206.5	$108,843.0	$202,356.0
>= 70	3518	4.3	21410	43000	$70,627.5	$373,541.0	$130,268.5	$668,607.0	$1,243,044.0
Total	40768	0.5	20175	5000	$8,212.5	$43,435.0	$15,147.5	$77,745.0	$144,540.0

For peer review only - http://bmjopen.bmj.com/site/about/guidelines.xhtml

Single Risk Factor Approach: BP ≥ 140/90 (SBP ≥ 140 + isolated raised DBP), %

	Total no. of people in the general population (in thousands)*	Percentage of population aged ≥40 years requiring medication (%)	Total no. of people estimated to require drug treatment (in thousands)	No. of people per million population (aged 40 years and older)	Aspirin	Enalapril	Hydrochlorothiazide	Simvastatin	Total
Age Group									
Men									
40 to 49	9210	17.1	156482	171000	$280,867.5	$1,485,477.0	$518,044.5	$2,658,879.0	$4,943,268.0
50 to 59	6303	21.7	129375	217000	$356,422.5	$1,885,079.0	$657,401.5	$3,374,133.0	$6,273,036.0
60 to 69	3730	30.5	96228	305000	$500,962.5	$2,649,535.0	$923,997.5	$4,742,445.0	$8,816,940.0
>= 70	1881	29.4	73529	294000	$482,895.0	$2,553,978.0	$890,673.0	$4,571,406.0	$8,498,952.0
Total	21124	22.1	458995	221000	$362,992.5	$1,919,827.0	$669,519.5	$3,436,329.0	$6,388,668.0
Women									
40 to 49	9087	22.5	199643	225000	$369,562.5	$1,954,575.0	$681,637.5	$3,498,525.0	$6,504,300.0
50 to 59	5662	30.6	164475	306000	$502,605.0	$2,658,222.0	$927,027.0	$4,757,994.0	$8,845,848.0
60 to 69	3257	28.7	81881	287000	$471,397.5	$2,493,169.0	$869,466.5	$4,462,563.0	$8,296,596.0
>= 70	1638	33.7	83509	337000	$553,522.5	$2,927,519.0	$1,020,941.5	$5,240,013.0	$9,741,996.0
Total	19644	26.7	522759	267000	$438,547.5	$2,319,429.0	$808,876.5	$4,151,583.0	$7,718,436.0
All									
40 to 49	18296	20.2	364085	202000	$331,785.0	$1,754,774.0	$611,959.0	$3,140,898.0	$5,839,416.0
50 to 59	11965	26.3	298189	263000	$431,977.5	$2,284,681.0	$796,758.5	$4,089,387.0	$7,602,804.0
60 to 69	6989	29.5	177236	295000	$484,537.5	$2,562,665.0	$893,702.5	$4,586,955.0	$8,527,860.0
>= 70	3518	31.6	157336	316000	$519,030.0	$2,745,092.0	$957,322.0	$4,913,484.0	$9,134,928.0
Total	40768	24.6	992585	246000	$404,055.0	$2,137,002.0	$745,257.0	$3,825,054.0	$7,111,368.0

For peer review only - http://bmjopen.bmj.com/site/about/guidelines.xhtml

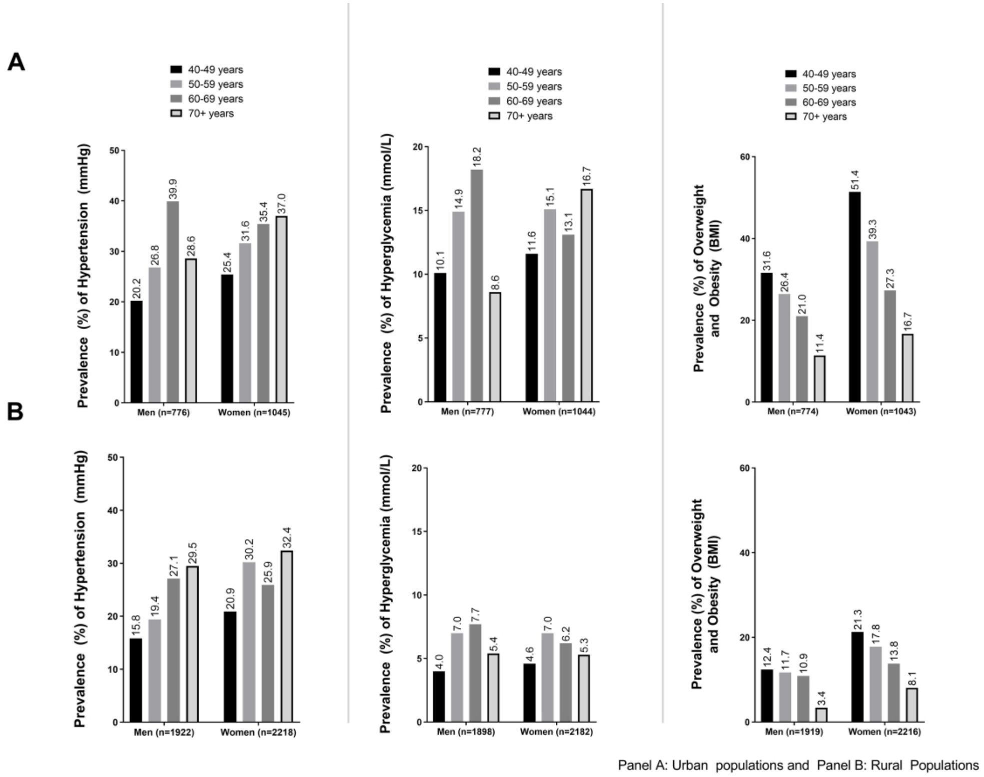

Figure 1: Prevalence of hypertension, hyperglycemia, and overweight and obesity among Bangladeshi adults aged 40 years and above by area of residence and age group.

281x220mm (300 x 300 DPI)

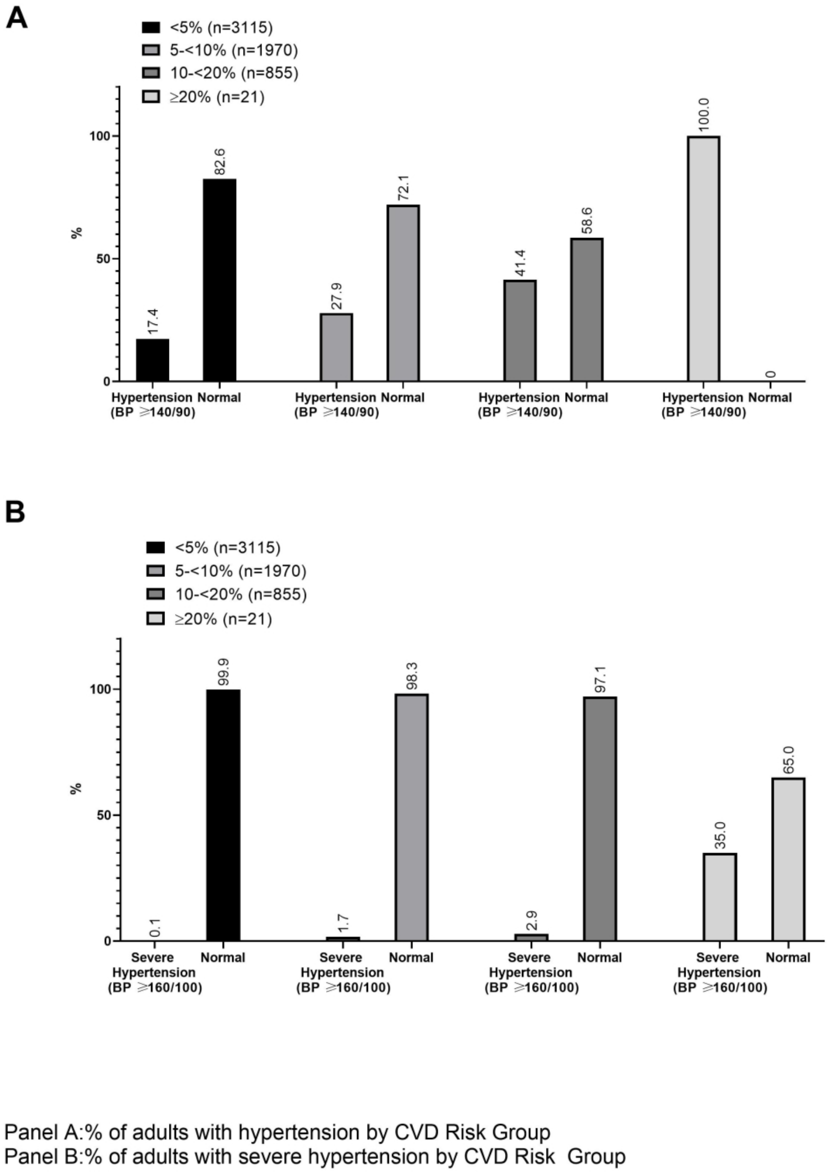

Figure 2: Proportion of adults with hypertension or severe hypertension by CVD risk group

205x276mm (300 x 300 DPI)

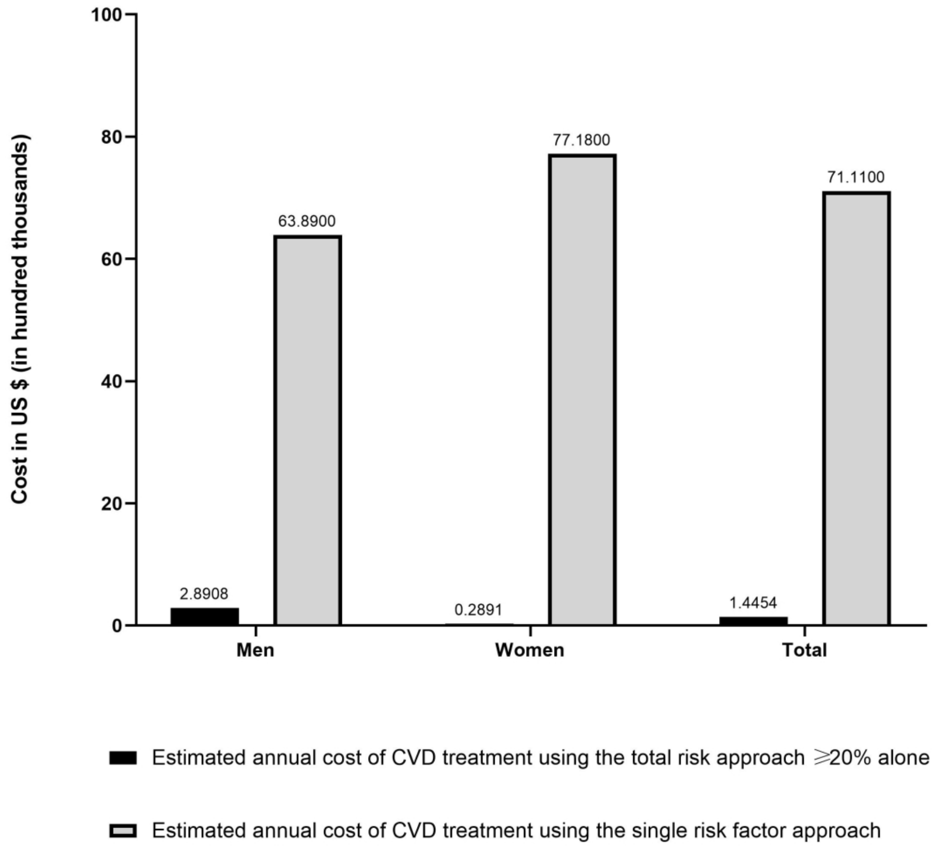

Figure 3: Annual costs of pharmacologic treatment for CVD by sex and risk stratification approach

199x175mm (300 x 300 DPI)

Appendix Table 1: Cost of common drugs used to treat cardiovascular disease in Bangladesh (converted to US$)

Drug Name	Dose	Number of Tablets	Category	Median price (in Bangladeshi Taka)	Price (in US$)*
Aspirin	100 mg	100 tablets	Antiplatelet drugs	38BDT	0.45
Atenolol	50 mg	100 tablets	Antihypertensive, Beta-adrenoreceptor blocking drugs	77BDT	0.92
Captopril	25 mg	100 tablets	Antihypertensive, ACE inhibitors	300 BDT	3.58
Chrlorthalidone	25 mg	30 tablets	Antihypertensive, thiazide diuretics	60 BDT	0.72
Enalapril	10 mg	100 tablets	Antihypertensive, ACE inhibitors	200 BDT	2.38
Frusemide	40 mg	100 tablets	Antihypertensive, loop diuretics	53 BDT	0.63
Hydrochlorothiazide	25 mg	100 tablets	Antihypertensive, thiazide diuretics	70 BDT	0.83
Isosorbide dinitrate	10 mg	100 tablets	Angina treatment, nitrates - coronary vasodilators	35 BDT	0.42
Labetalol	100 mg	60 tablets	Antihypertensive, Beta-adrenoreceptor blocking drugs	361.2 BDT	4.31
Losartan	50 mg	100 tablets	Antihypertensive, Angiotensin-II receptor blocker	600 BDT	7.15
Lovastatine	20 mg	30 tablets	Lipid regulation, statin	303.3 BDT	3.62
Methyldopa	250 mg	100 tables	Antihypertensive, Centrally acting or central sympatholytic	308 BDT	3.67
Nifedipine (slow release)	20 mg	100 tablets	Angina treatment, calcium-channel blocker	64 BDT	0.76
Propranolol hydrochloride	10 mg	100 tablets	Antihypertensive, Beta-adrenoreceptor blocking drugs	24 BDT	0.29
Simvastatine	10 mg	30 tablets	Lipid regulation, statin	360 BDT	4.29

For peer review only

Spironolactone	25 mg	100 tablets	Antihypertensive, potassium-sparing diuretics & aldoesterone antagonist	202 BDT	2.41
Streptokinase	1.5 million unit/vial	One vial	Anticoagulant	3100 BDT	36.96
Warfarin	5 mg	100 tablets	Anticoagulant	300 BDT	3.58

Abbreviations: mg, milligrams; US, United States; BDT, Bangladeshi Taka

*Price conversion based on exchange rate on 02/07/2019

For peer review only

STROBE 2007 (v4) Statement—Checklist of items that should be included in reports of *cross-sectional studies*

Section/Topic	Item #	Recommendation	Reported on page #
**Title and abstract**	1	(*a*) Indicate the study's design with a commonly used term in the title or the abstract	2
		(*b*) Provide in the abstract an informative and balanced summary of what was done and what was found	2-3
**Introduction**			
Background/rationale	2	Explain the scientific background and rationale for the investigation being reported	5-6
Objectives	3	State specific objectives, including any prespecified hypotheses	6
**Methods**			
Study design	4	Present key elements of study design early in the paper	6
Setting	5	Describe the setting, locations, and relevant dates, including periods of recruitment, exposure, follow-up, and data collection	7
Participants	6	(*a*) Give the eligibility criteria, and the sources and methods of selection of participants	6
Variables	7	Clearly define all outcomes, exposures, predictors, potential confounders, and effect modifiers. Give diagnostic criteria, if applicable	8-10
Data sources/ measurement	8*	For each variable of interest, give sources of data and details of methods of assessment (measurement). Describe comparability of assessment methods if there is more than one group	7-10
Bias	9	Describe any efforts to address potential sources of bias	6-7 & 15-16
Study size	10	Explain how the study size was arrived at	7
Quantitative variables	11	Explain how quantitative variables were handled in the analyses. If applicable, describe which groupings were chosen and why	9-10
Statistical methods	12	(*a*) Describe all statistical methods, including those used to control for confounding	9-10
		(*b*) Describe any methods used to examine subgroups and interactions	8-11
		(*c*) Explain how missing data were addressed	9
		(*d*) If applicable, describe analytical methods taking account of sampling strategy	N/A
		(*e*) Describe any sensitivity analyses	N/A
**Results**			

For peer review only

Participants	13*	(a) Report numbers of individuals at each stage of study—eg numbers potentially eligible, examined for eligibility, confirmed eligible, included in the study, completing follow-up, and analysed	10
		(b) Give reasons for non-participation at each stage	N/A
		(c) Consider use of a flow diagram	N/A
Descriptive data	14*	(a) Give characteristics of study participants (eg demographic, clinical, social) and information on exposures and potential confounders	10
		(b) Indicate number of participants with missing data for each variable of interest	9
Outcome data	15*	Report numbers of outcome events or summary measures	
Main results	16	(*a*) Give unadjusted estimates and, if applicable, confounder-adjusted estimates and their precision (eg, 95% confidence interval). Make clear which confounders were adjusted for and why they were included	N/A
		(*b*) Report category boundaries when continuous variables were categorized	N/A
		(*c*) If relevant, consider translating estimates of relative risk into absolute risk for a meaningful time period	N/A
Other analyses	17	Report other analyses done—eg analyses of subgroups and interactions, and sensitivity analyses	10
**Discussion**			
Key results	18	Summarise key results with reference to study objectives	12-14
Limitations	19	Discuss limitations of the study, taking into account sources of5potential bias or imprecision. Discuss both direction and magnitude of any potential bias	15-16
Interpretation	20	Give a cautious overall interpretation of results considering objectives, limitations, multiplicity of analyses, results from similar studies, and other relevant evidence	13-16
Generalisability	21	Discuss the generalisability (external validity) of the study results	15
**Other information**			
Funding	22	Give the source of funding and the role of the funders for the present study and, if applicable, for the original study on which the present article is based	19

*Give information separately for cases and controls in case-control studies and, if applicable, for exposed and unexposed groups in cohort and cross-sectional studies.

Note: An Explanation and Elaboration article discusses each checklist item and gives methodological background and published examples of transparent reporting. The STROBE checklist is best used in conjunction with this article (freely available on the Web sites of PLoS Medicine at http://www.plosmedicine.org/, Annals of Internal Medicine at http://www.annals.org/, and Epidemiology at http://www.epidem.com/). Information on the STROBE Initiative is available at www.strobe-statement.org.

For peer review only

BMJ Open

Estimation of total cardiovascular risk using the 2019 WHO CVD prediction charts and comparison of population-level costs based on alternative drug therapy guidelines: a population-based study of adults in Bangladesh

Journal:	*BMJ Open*
Manuscript ID	bmjopen-2019-035842.R2
Article Type:	Original research
Date Submitted by the Author:	21-Apr-2020
Complete List of Authors:	Islam, Jessica; World Health Organization Country Office for Bangladesh, ; University of North Carolina at Chapel Hill Gillings School of Global Public Health, Department of Epidemiology<br>Zaman, MM; World Health Organization, Dhaka, Bangladesh,<br>Moniruzzaman, Mohammad; Shiga University of Medical Science, Public Health<br>Ara Shakoor, Shawkat; National Institute of Opthalmology<br>Hossain, A.H.M. Enayet; National Institute of Opthalmology
**Primary Subject Heading**:	Epidemiology
Secondary Subject Heading:	Cardiovascular medicine
Keywords:	Cardiac Epidemiology < CARDIOLOGY, EPIDEMIOLOGY, Hypertension < CARDIOLOGY, Ischaemic heart disease < CARDIOLOGY, Myocardial infarction < CARDIOLOGY

SCHOLARONE™
Manuscripts

I, the Submitting Author has the right to grant and does grant on behalf of all authors of the Work (as defined in the below author licence), an exclusive licence and/or a non-exclusive licence for contributions from authors who are: i) UK Crown employees; ii) where BMJ has agreed a CC-BY licence shall apply, and/or iii) in accordance with the terms applicable for US Federal Government officers or employees acting as part of their official duties; on a worldwide, perpetual, irrevocable, royalty-free basis to BMJ Publishing Group Ltd ("BMJ") its licensees and where the relevant Journal is co-owned by BMJ to the co-owners of the Journal, to publish the Work in this journal and any other BMJ products and to exploit all rights, as set out in our licence.

The Submitting Author accepts and understands that any supply made under these terms is made by BMJ to the Submitting Author unless you are acting as an employee on behalf of your employer or a postgraduate student of an affiliated institution which is paying any applicable article publishing charge ("APC") for Open Access articles. Where the Submitting Author wishes to make the Work available on an Open Access basis (and intends to pay the relevant APC), the terms of reuse of such Open Access shall be governed by a Creative Commons licence – details of these licences and which Creative Commons licence will apply to this Work are set out in our licence referred to above.

Other than as permitted in any relevant BMJ Author's Self Archiving Policies, I confirm this Work has not been accepted for publication elsewhere, is not being considered for publication elsewhere and does not duplicate material already published. I confirm all authors consent to publication of this Work and authorise the granting of this licence.

For peer review only

Estimation of total cardiovascular risk using the 2019 WHO CVD prediction charts and comparison of population-level costs based on alternative drug therapy guidelines: a population-based study of adults in Bangladesh

Running title: Total cardiovascular risk approach in Bangladesh

Authors: Jessica Yasmine Islam[1], M Mostafa Zaman[2], Mohammad Moniruzzaman[3], Shawkat Ara Shakoor[4], A.H.M. Enayet Hossain[4]

Author Affiliations:

[1]Department of Epidemiology, Gillings School of Global Public Health, University of North Carolina at Chapel Hill, North Carolina, USA

[2]Research and Publication Unit, World Health Organization, Dhaka, Bangladesh

[3]Center for Epidemiologic Research in Asia, Shiga University of Medical Science, Otsu, Shiga, Japan

[4]National Institute of Ophthalmology. Sher-e-Bangla Nagar, Dhaka, Bangladesh

Corresponding Author:

Dr. Jessica Yasmine Islam, PhD, MPH
Department of Epidemiology
UNC Gillings School of Global Public Health
UNC Chapel Hill
135 Dauer Drive
Chapel Hill, NC 27599
Email: islamjy@email.unc.edu

For peer review only

Abstract

Objective: The objective of this study is to estimate the population distribution of 10-year cardiovascular disease (CVD) risk among Bangladeshi adults aged 40 years and above, using the 2019 World Health Organization (WHO) CVD Risk Prediction Charts. Additionally, we compared the cost of CVD pharmacologic treatment based on the total CVD risk (thresholds ≥ 30%/ ≥ 20%) and the single risk factor (hypertension) cutoff levels in the Bangladeshi context.

Study Design: Cross-sectional, population-based study

Setting and Participants: From 2013-2014, we collected data from a nationally representative cross-sectional survey of adults aged ≥40 years from urban and rural areas of Bangladesh (n = 6189). We estimated CVD risk using the 2019 WHO CVD Risk Prediction Charts and categorized as very low (<5%), low (5-<10%), moderate (10-<20%), high (20-<30%), and very high risk (≥30%). We estimated drug therapy costs using the lowest price of each drug class available (aspirin, thiazide diuretics, statins, and angiotensin-converting enzyme inhibitors). We compared the total cost of drug therapy using the total CVD risk vs. single risk factor approach.

Primary Outcome Measures: Our primary outcome was 10-year CVD risk categorized as very low (<5%), low (5-<10%), moderate (10-<20%), high (20-<30%), and very high risk (≥30%).

Results: The majority of adults (85.2%, 95% CI: 84.3–86.1) have a 10-year CVD risk of less than 10%. The proportion of adults with a 10-year CVD risk of ≥20% was 0.51%. Only one adult was categorized with a 10-year CVD risk of ≥30%. Among adults with CVD risk groups of very low, low, and moderate, 17.4%, 27.9%, and 41.4% had hypertension (BP ≥140/90) and 0.1%, 1.7%, and 2.9% had severe

For peer review only - http://bmjopen.bmj.com/site/about/guidelines.xhtml

hypertension (BP≥160/100), respectively. Using the total CVD risk approach would reduce drug costs per million populations to $144,540 (risk of ≥ 20%).

Conclusion: To reduce health care expenditure for the prevention and treatment of CVD, a total risk approach using the 2019 WHO CVD Risk Prediction Chart may lead to cost-savings.

Keywords: non-communicable diseases, Bangladesh, cardiovascular disease, pharmacologic intervention, cost, chronic disease

For peer review only

For peer review only - http://bmjopen.bmj.com/site/about/guidelines.xhtml

Article Summary

Strengths and Limitations of the Study

- Using the recently updated 2019 WHO CVD Risk Prediction Charts, this study provides evidence for incorporating the WHO CVD Risk Prediction Charts into CVD management and health care guidelines, and may lead to potential cost-savings from a societal perspective.
- The 2019 WHO CVD Risk Prediction Charts should be applied to a population who have not experienced a CVD event in the past, however, we were unable to confirm self-reported medical history of participants using medical charts or health records, leading to the potential for measurement error due to recall bias.
- The cost estimates we present are an underestimate of total costs for CVD-related treatment as the focus of this study is on the cost of pharmacologic intervention only as the largest contributor to overall direct costs in Bangladesh.
- Although we present the total number of people estimated to require drug treatment using 2014 population data, we were unable to identify population estimates of only those at risk of their first CVD event due to lack of surveillance data.
- The CVD 10-year risk cut-offs were defined using risk prediction models derived from 85 cohorts mostly from high-income countries, as data from large-scale prospective cohort data from most low-income and middle-income countries (LMICs) are limited.

For peer review only - http://bmjopen.bmj.com/site/about/guidelines.xhtml

Background

Globally, cardiovascular disease (CVD) is the leading cause of death and disproportionately impacts low- and middle-income countries (LMICs), where over 75% of CVD-related deaths occur (1). People living in LMICs are at high risk of developing CVDs due to the absence of integrated primary care for early detection and prevention of CVD-related risk factors, such as high cholesterol, high blood pressure, and smoking. Limited access to primary care and the growing burden of CVDs is a significant cause of poverty in LMICs and hinders the macroeconomic development of many countries(2). LMICs are estimated to experience cumulative economic losses exceeding 7 trillion US dollars over the next 15 years due to morbidity and mortality caused by noncommunicable diseases, including CVD(3). As such, the significance of the CVD epidemic has gained increasing international recognition over the past decade, leading to the development of several international guidelines for CVD control and prevention(4).

In 2007, the World Health Organization (WHO) published pocket guidelines, including CVD risk prediction charts, designed for health care workers in LMICs to guide patient 10-year risk stratification for heart attack or stroke(5). There are two possible strategies suitable for a low resource setting to assess the risk of a cardiovascular event and identify those at high risk of a fatal CVD outcome: 1. Utilize a single risk factor management strategy, which focuses on one condition at a time, such as hypertension; or, 2. Utilize a more holistic approach considering several risk factors such as age, tobacco use, gender, diabetes diagnosis, body mass index, blood pressure, and blood cholesterol when measured. Through the total risk approach, pocket guidelines help to identify high-risk patients that are in imminent danger of a heart attack or stroke for timely pharmacologic treatment or surgical interventions. Additionally, applying the total risk approach via the WHO prediction charts in a nationally representative sample provides an opportunity to estimate and monitor the population-level distribution of CVD risk to ultimately inform CVD treatment policy recommendations (6, 7).

Currently, Bangladesh has not incorporated clinical guidelines for screening or treatment of risk factors based on absolute CVD risk scores and estimation of the population distribution of CVD risk over time. Data are needed to support the implementation of the WHO prediction charts as clinical guidelines

For peer review only - http://bmjopen.bmj.com/site/about/guidelines.xhtml

for Bangladesh, a resource-limited setting, to demonstrate the potential cost-savings and benefit of the WHO recommendation on CVD prevention. Recently in 2019, the WHO updated the CVD risk charts based on newly validated risk prediction models to estimate CVD risk in 21 Global Burden of Disease regions(8). The newly developed risk prediction models have been calibrated using data from The Global Burden of Disease study to include estimates from LMICs. To our knowledge, here we present the first analysis to apply the updated CVD risk charts among a cohort of Bangladeshi adults. Prior studies conducted in Bangladesh have estimated CVD risk among adults residing in rural areas only and have not included a nationally representative population (9-11). Additionally, no prior studies have estimated the potential costs of pharmacological treatment for CVD in Bangladesh using either the single risk factor or total CVD risk approach, as done previously in other settings(7). Our objective was to assess the distribution of absolute CVD risk among a nationally representative sample of Bangladeshi adults using the 2019 WHO CVD Risk Prediction Chart recommended for the WHO South Asian Region (Bangladesh, Bhutan, India, Nepal, and Pakistan). We also compared the costs of drug treatments for CVD prevention using the total cardiovascular risk thresholds at ≥20% and with single risk factor cutoff levels (blood pressure ≥ 140/90 mm Hg).

Methods

Study design and setting

Data were analyzed from a population-based cross-sectional study conducted from September to December 2013 to assess the burden of blindness and low vision among adults in Bangladesh. The target population of this survey included men and women residing in Bangladesh over the age of 40 years. The exclusion criteria included tourists and the institutionalized, such as residents of a military base, hospital, prisons, nursing homes, and other such institutions. We obtained ethical approval for this study from the Institutional Review Board of the National Institute of Ophthalmology. We provided participants with detailed information regarding the study objectives and procedures using a printed handout prepared in

For peer review only - http://bmjopen.bmj.com/site/about/guidelines.xhtml

Bengali. Written consent was obtained from participants through signature or, if not possible through thumbprint.

Sampling Frame

We adopted a multistage, geographically clustered, probability-based sampling approach to obtain a nationally representative sample of Bangladesh, as previously described and outlined per the WHO STEPwise approach (12-15). Population statistics were obtained using the 2011 national census conducted by the Bangladesh Bureau of Statistics (BBS) to create the sample frame (16). The sampling frame included 64,407 primary sampling units (PSUs) covering all seven divisions of the country. We randomly selected seventy-two PSUs (25 urban and 47 rural) from seven divisions, with the probability of selection proportional to the population size of each division. In each PSU, we selected 100 consecutive households as the secondary sampling unit.

For each household, a trained field data collector approached the head of the household or the family member most knowledgeable of the residents to screen for eligible participants. The screening respondent was asked to describe the composition of household residents, which was defined as those who considered the home to be their primary place of residence as of the night before. A list was composed and ordered from youngest to oldest age in years starting from 40 years. Using the list of eligible residents, we used the Kish table approach to randomly select one participant from each home. The selected participant was asked to come to a nearby health center the next day to administer the survey by trained study interviewers and undergo a medical examination by the study physician. Based on the medical review, participants were followed-up with by the providers at the health center for treatment.

Patient and Public Involvement

There was no patient or public involvement in the implementation of this study or interpretation of analytic results.

For peer review only - http://bmjopen.bmj.com/site/about/guidelines.xhtml

Data Collection

To ensure effective and uniform data collection, field interviewers underwent a seven-day training on the interview methodology by the study ophthalmologists and epidemiologists. The training included an in-depth review of the survey content and protocol for completing the demographic questionnaire (a modified WHO/PBL Version III). Each member of the data collection team was provided a detailed survey protocol manual outlining the survey activities, the questionnaire interview, and information about the duties and responsibilities of all survey personnel.

Demographic data were collected, including age, sex, marital status, educational level, and occupation, using a structured questionnaire survey. Data regarding tobacco use, health history, and treatment history were also collected. Participants were asked if they smoked (e.g. cigarette, hookah, pipe) or if they used smokeless tobacco (e.g. chewing tobacco, jorda) to assess the history of tobacco use. Each participant provided medical history for a prior diagnosis of high blood pressure or hypertension, diabetes, renal disease, or any cardiovascular diseases by a health care provider. Medication history was obtained including medication for high blood pressure, diabetes, malaria, steroids, tuberculosis, and among women, history of oral contraception. The questionnaire was translated from English to Bengali, adapted, and validated before data collection.

Physical measurements, including height, weight, and blood pressure (BP) were collected. Trained field interviewers measured BP using an appropriately calibrated aneroid sphygmomanometer with appropriately sized arm cuffs. BP measurements were consistently taken on the right arm at heart level and elbow-assisted while the participant was seated. The initial measurement was performed after five minutes of rest. After two minutes, the second measurement was taken. The mean of these two BP readings was utilized as the final BP for each participant. To measure blood glucose levels, we obtained random blood glucose samples (17). Capillary blood samples were consistently taken using the right arm and index finger with a glucometer (Accu-chek Advantage, Roche Diagnostics Division, Grenzacherstrasse, Switzerland).

For peer review only - http://bmjopen.bmj.com/site/about/guidelines.xhtml

Estimation of 10-year CVD risk

We estimated 10-year CVD risk using the 2019 WHO CVD risk prediction charts(8, 18). The prediction charts provide the 10-year risk of a fatal or non-fatal major cardiovascular event, such as myocardial infarction or stroke, based on age, sex, blood pressure, body mass index, smoking status, total blood cholesterol, and the presence or absence of diabetes mellitus for 14 WHO epidemiological sub-regions. For each region, two sets of charts have been developed based on the availability of laboratory-based results. As total cholesterol was not measured in our cohort, we utilized the WHO cardiovascular disease risk non-laboratory-based charts developed for South Asia (including, Bangladesh, Bhutan, India, Nepal, and Pakistan). The non-laboratory-based risk charts do not account for diabetes diagnosis or total cholesterol levels.

The prediction chart grades CVD risk using the following categories: age (1: 40–44years; 2: 45-49 years; 3: 50-54 years; 4: 55-59 years; 5: 60-64 years; 6: 64-69 years; 7: 70-74 years), sex (men and women), smoking (smoker or non-smoker), systolic blood pressure (<120 mmHg, 120 to 139, 140 to 159, 160 - <180, and ≥180), and body mass index (<20, 20-24, 25-29, 30-35, and ≥35). The risk categories for 10-year combined acute myocardial infarction and stroke (fatal and non-fatal) are as follows: <5%, 5-<10%, 10 - <20%, 20 to <30%, and ≥ 30%.

Observations with missing values were dropped from the analysis. We did not anticipate any bias from the complete-case analysis approach as the number of missing observations for key variables was less than 2%: smoking status, n=18 or 0.3% missing values; systolic blood pressure, n = 22 or 0.4% missing values; diastolic blood pressure, n = 25 or 0.4% missing values; and body mass index, n=30 or 0.5%,

Data Analysis

We present sociodemographic variables using mean (standard deviation) or the median (interquartile range) for continuous variables and proportion for categorical variables. We conducted bivariate analyses by sex and age group. We used the chi-square test to assess for any significant

For peer review only - http://bmjopen.bmj.com/site/about/guidelines.xhtml

differences in CVD risk distribution across sex. For estimating the cost of medicines per million per year (population aged 40 years or older), we used the lowest price of each drug class available in the market (generic preparation of aspirin, thiazide diuretics, statins, and angiotensin-converting enzyme inhibitors). Appendix Table 1 includes further details regarding the specific costs of common drugs used to treat cardiovascular disease in Bangladesh.

T0 calculate costs we included the following categories: (1) people with high cardiovascular risk (≥20% and BP ≥160/100), who are recommended for pharmacological intervention using four different types of drugs for treatment(5, 7); and (2) people with BP ≥140/90, who are recommended antihypertensive treatment. To calculate the estimated annual total cost of CVD medication treatment per million populations (aged 40 years or older), we multiplied the percentage of the population at risk and the price of medicine in Bangladesh. We included an estimate of the total number of people estimated to require drug treatment as follows: we multiplied the prevalence of the population requiring medication based on each approach by the number of people in the general population in 2013 (19) stratified by gender and age group.

Results

Demographic Characteristics

The mean age of included participants was 52.9 years (men: 53.5 years, women: 52.5 years) (Table 1). The average level of educational attainment was 3.1 years of education; women were generally less educated than men (2.1 years vs. 4.3 years, respectively). The majority (80%) of women were housewives, and among men, the most common occupation was an industrial worker or day laborer (51.2%). Overall, over one-third of participants ever used smoking tobacco, and over half ever used smokeless tobacco. Few participants drank alcohol in the past 30 days (1.2%). The mean BMI was 21.9 $kg/m^{2,}$ and the mean waist circumference was 82.4 cm.

Overall, the prevalence of hypertension (defined as BP ≥140/90) increased with age among men and women. Additionally, women had a higher prevalence of hypertension among nearly all age-groups

For peer review only - http://bmjopen.bmj.com/site/about/guidelines.xhtml

in both urban and rural areas. The prevalence of hyperglycemia (glucose ≥11.1 mmol/L) was higher among urban adults compared to rural across all age groups. The highest prevalence of hyperglycemia was observed among urban men aged 60-69 years at 18.2%. Finally, the prevalence of overweight and obesity was higher among urban residents than rural residents. The largest prevalence of overweight and obesity was observed among urban women, with prevalence as high as 51.4% among women aged 40-49 years (Figure 1).

For peer review only

Distribution of Cardiovascular Risk

We summarized the distribution of CVD risk in the population overall and stratified by sex in Table 2. Eighty-five percent of participants had a low (< 10%) 10-year CVD risk, and this proportion was significantly different across sex ($p<0.001$). Over half (63.7%) of women had a very low (<5%) cardiovascular risk. Almost all (99.5%) of the study population were categorized as having cardiovascular risk < 20%. A higher proportion of men (1.0%) were categorized as high risk than women (0.1%) ($p = <0.001$). Overall, only one male participant was categorized as very high risk or with a CVD risk of ≥ 30%.

We summarized the prevalence of adults with hypertension by CVD risk group (Figure 2). Among those with 10-<20% CVD risk, we observed a high proportion of hypertensive (41.4%). In the high-risk group (≥20%), 100% had hypertension. Additionally, among those with ≥20% CVD risk, we observed that 35% had severe hypertension (BP ≥160/100).

Cost of Drug Treatment

We were unable to compare the costs of drug treatment at two cardiovascular risk thresholds (30% to 20%) due to only one male adult with a CVD risk at ≥30%. We observed a low proportion of adults with CVD risk ≥20% at 0.5%. When we included BP ≥160/100 measurements, the number of people requiring treatment more than tripled from 0.5% to 1.8% (Table 3). Conversely, if a single risk factor approach was applied, and all those with hypertension (a persistent SBP ≥140 and/or DBP ≥90)

For peer review only - http://bmjopen.bmj.com/site/about/guidelines.xhtml

were treated,24.6%of the sample would require drug treatment, specifically antihypertensive; more than 20 times the proportion identified when using the total cardiovascular risk approach alone.

Comparison of Cost by Approach

Next, we compared the estimated annual cost of medicines per million populations for implementing the total risk approach vs. the single risk factor approach. Table 4 shows the estimated number of people aged 40 years or older requiring drug treatment stratified by age group and gender. The estimates showed that if the single risk factor approach is applied in Bangladesh with its percentage of the population at risk and the lowest price of medicine in the country, the cost per million populations (aged 40 years or older) of treating those with BP ≥140/90 would be $7,111,368 US$; if the absolute risk approach were applied, the cost of treating those with a 10-year risk of CVD ≥20% per million populations (aged 40 years or older) would be $144,540, almost fifty times less (Figure 3). The cost estimation was based on the percentage of the population at different levels of risk and the differences in the price of generic medicines. For this analysis, we focused on the cost of pharmacological treatment as it is the most critical contributor to the overall direct costs of CVD treatment in Bangladesh. We assumed that other costs of CVD treatment and prevention service delivery, such as health facilities and wages of health workers, are similar for both approaches of service delivery(7).

Discussion

Using this nationally representative survey of Bangladeshi adults aged 40 years and above, we found that the majority of adults (97%) were at a very low, low or moderate ten-year risk of myocardial infarction and stroke. The proportion of adults requiring drug treatment rose from 1.0% to 2.1% when the threshold for pharmacologic intervention was changed from ≥20% alone to ≥20% plus blood pressure of 160/100, respectively; which was lower the proportion than the single risk factor approach (24.6%). Our data demonstrate that using a single risk factor approach to manage individual cardiovascular risk factors is costlier ($7,111,368per million population) than using the total risk approach (CVD risk ≥20, $144,540

For peer review only - http://bmjopen.bmj.com/site/about/guidelines.xhtml

per million population), as a more substantial proportion of adults will need drug treatment. Findings from this analysis support the implementation of clinical guidelines using CVD risk scores calculated using the WHO CVD Risk Prediction Charts to appropriately identify patients at the highest risk of CVD development over ten years in Bangladesh.

In our study using nationally representative data, we found that the 10-year risk of CVD was low (<10%) among the vast majority of adults (85.1%). Additionally, only 0.5% of adults were at high risk (≥ 20%) of a CVD event within the next ten years. In this analysis, we applied the 2019 CVD Risk Charts for South Asia, which are newly developed and now incorporate body mass index as part of the prediction chart algorithm. Our results are comparable to regional data presented in the 2019 Lancet publication by the WHO CVD Risk Chart Working Group, which showed that 0% of women from Bhutan, Sri Lanka, and Nepal had a CVD risk level above 20%. Similarly, 0% of men from Bhutan, and only <2% of men from both Sri Lanka and Nepal were categorized with a risk level above 20%(8). These data demonstrate a lower prevalence of CVD risk ≥20% than prior reports from South Asia, which utilized the original risk prediction charts published in 2007. For example, prior data from Nepal (20) and Sri Lanka(21) showed that 4.3% and 8.2% of adults respectively, were categorized with a high (≥20%) 10-year risk of a CVD event. Further, analyses from a rural area of South India revealed that seventeen percent of participants had moderate to high risk (10->20%) of cardiovascular events per the 2007 WHO prediction charts(22). Finally, data collected in 2010 from Pakistan showed that 10% of adults were categorized with ≥20% CVD risk, with 2.9% as high as ≥40%(7). When utilizing the 2019 WHO prediction charts on the population level to measure and monitor trends in total CVD risk in recent years, policy makers in LMICs should interpret the trends with caution, and assess changes in trends of CVD-risk over time using both the 2007 and 2019 WHO risk prediction charts for comparison.

The WHO CVD Risk Charts were developed for use in LMICs and are now more suitable for use in these settings due to the inclusion of data from low-resource regions in the risk prediction model development. While we present the first country-specific analysis using the 2019 risk charts, several prior studies in LMICs have been conducted using the 2007 risk charts. In other countries in Asia, we observe

For peer review only - http://bmjopen.bmj.com/site/about/guidelines.xhtml

the prevalence of "high CVD risk" (≥20%) of 6.0%, 2.3% and 1.3% in Mongolia, Malaysia and Cambodia, respectively(23). Mendis *et al* reported the 10 year CVD risk of seven countries and the majority of these countries reported low CVD risk among its adult populations [China (96.1%) and Sri Lanka (94.9%), (Iran (93.9%), Cuba (89.7%), Nigeria (86.0%), Georgia (83.1%), and Pakistan (79.2%)](7). Studies conducted in urban areas of low- and middle-income countries show varying prevalence of high CVD risk (≥ 20%): for example, one study from Malaysia shows 20.5% of adults were high-risk of CVD(24), whereas studies from urban Kenya(25) and Sri Lanka(21) reports less than 10% of their population had high CVD risk._ Specifically in Bangladesh, utilizing the 2007 prediction charts, three prior studies have reported absolute CVD risk among the adult population (10, 26, 27). Fatema et al. found that 10% of rural Bangladeshi adults were at high risk (≥ 20%) of a CVD event within the next ten years, and half of these adults fell in the very high-risk category (≥ 30%). In another rural Bangladeshi population, the proportion of participants at a high-risk (≥ 20%) of a CVD event was 2.1% (26). Finally, in an urban Bangladeshi population of 150 adults, 3.4% had high CVD risk (≥ 20%), which is lower than expected as the population was urban(27). No other studies in Bangladesh have been conducted to assess the 10-year risk of CVD using the WHO CVD Risk Prediction Charts. We present novel data using the 2019 charts and a nationally representative sample, which may be generalizable to the population of Bangladesh. Data we present may be used to inform policymakers decisions on clinical guidelines and resource allocation for treatment of CVDs in Bangladesh.

Similar to prior analyses conducted using data from eight LMICs (7), our results demonstrate that in the Bangladeshi context using a single risk factor approach to evaluate the risk of CVD-related mortality would cost more than implementing the total risk approach due to higher drug costs. In Bangladesh, about 60% of out-of-pocket costs patients face goes towards drugs directly bought from pharmacies, diagnostics, and informal providers(28). Currently, Bangladesh does not offer universal health coverage or affordable health insurance plans. The cost of treatment for CVD frequently leads to catastrophic health expenditure and impoverishment; the proportion of catastrophic spending for treatment is highest among those from the lowest quintiles of wealth (14%) compared to those with high

For peer review only - http://bmjopen.bmj.com/site/about/guidelines.xhtml

wealth and high socioeconomic status (6.6%) (29). As such, implementing the WHO CVD Risk Prediction Charts may be beneficial to patients in Bangladesh as only those at the highest risk of future CVD would be recommended for treatment.

In addition to benefits to the patient, the total CVD risk approach would also be beneficial to Bangladesh's health care system by improving NCD preventive service delivery and the use of guidelines for adequate care. Currently, Bangladesh is categorized by the World Bank as a lower-middle-income country with emerging health challenges as the burden of NCDs continues to grow. In 2015, an estimated 67% of all deaths in Bangladesh were due to NCDs and the risk of premature death from chronic disease among adults aged 30-70 years was 22%(30). Indeed, CVDs and circulatory diseases are the leading cause of mortality and morbidity in Bangladesh. Despite this substantial burden, preventive services for CVDs in Bangladesh are limited. In 2014, an estimated 16% of health care facilities across the country (i.e. hospitals, community clinics) had the resources to diagnose, prescribe treatment for, and manage patients with CVDs(31). District hospitals (95%), Upazila health complexes (81%), and private hospitals (77%) were more likely to provide services for cardiovascular diseases than other facilities. Only 10% of community clinics and maternal and child welfare centers, and 17% of union level facilities, which are the most accessible providers in rural areas, provided any cardiovascular services, and the services at these facilities were limited to the measurement of blood pressure or referrals(31). Among facilities with the capacity to offer services for CVD management, about only 20% utilized established guidelines for hypertension treatment and less than one-third had essential CVD medicines readily available on-site for patients(31). By integrating the WHO risk prediction charts into the national guidelines for management of hypertension and CVD prevention in Bangladesh, the proportion of facilities using established guidelines may increase as the charts are easy to implement, interpret, and access. Additionally, since only one-third of facilities have essential CVD medicines readily available, distributing pharmacologic treatment to those at highest risk of premature mortality due to CVD will be crucial.

Although the implementation of a total risk approach may lead to cost-savings, there are limitations to implementing the 2019 CVD Risk Prediction Charts. When compared to the single risk

For peer review only - http://bmjopen.bmj.com/site/about/guidelines.xhtml

factor approach, the WHO charts categorizes fewer individuals as high-risk and may delay the receipt of necessary life-saving treatment. For example, he prediction charts may underestimate CVD risk in certain categories of people such as those with persistent raised blood pressure ≥160/100 mmHg, blood cholesterol ≥8 mmol/L, or those suffering from diabetes with renal disease(5). Patients who may fall in these categories should be recommended for intensive lifestyle interventions, and appropriate drug therapy, however, the CVD prediction charts will erroneously deny treatment to these potentially high-risk adults.. In fact, the risk models used to develop the 2019 CVD risk charts may have underestimated CVD risk due to limitations in the population data used to estimate incidences: Data used to develop the predictions models likely included people already on cardiovascular disease prevention therapies, such as statins, which have led to an underestimate in CVD risk(8). In our study, we underscore the potential for underestimation of CVD risk by comparing the proportion of adults categorized as high risk (≥20% CVD risk) to those who would be diagnosed with hypertension (BP ≥ 140/60) and severe hypertension (BP ≥ 160/100). Additionally, we provided a graphical summary of common risk factors of CVD, including hypertension, hyperglycemia, and overweight and obesity. Despite our very low proportion of adults who would be recommended for treatment based on the risk prediction charts, we observed a high prevalence of these risk factors particularly in urban populations.

Limitations of this analytic approach should be considered when interpreting our results. The CVD 10-year risk cut-offs were defined using risk prediction models derived from 85 cohorts mostly from high-income countries, as data from large-scale prospective cohort data from most LMICs are unavailable. Data were used from the Global Burden of Disease (GBD) project to recalibrate the models to be representative of LMICs, however, the GBD data do not have country-specific disease risk estimates. As such, the estimation used from each region's chart will most likely apply to the largest country within each region, or from the country where most of the data originated. The risk prediction charts provide approximate estimates of CVD risk in people who do not have established coronary heart disease, stroke or other atherosclerotic diseases. Although we included simvastatin in our pharmacologic cost analysis, we were unable to measure total cholesterol or confirm the medical history of participants

For peer review only - http://bmjopen.bmj.com/site/about/guidelines.xhtml

using medical charts and relied on self-report, leading to the potential for measurement error and recall bias. Additionally, we were unable to categorize participants as diabetic as we did not obtain fasting blood glucose and were only able to categorize adults as hyperglycemic in our descriptive analysis as we measured random blood glucose. Further, our data were collected in 2013 and may be outdated as population growth in older age groups has been observed in recent years. Our analyses should be replicated using more recent data and future research studies should include the measurement of total cholesterol. Finally, our cost estimates were based on the prevalence of each risk approach in our study sample. Although we present the total number of people estimated to require drug treatment using 2013 population data, estimates of only those at risk of their first CVD event were unavailable due to lack of surveillance data. Nevertheless, our data are valuable as the first analysis to apply the 2019 WHO CVD risk prediction charts to a cohort of adults in Bangladesh. Additionally, we provide data on the comparative cost difference of each approach to underscore the potential cost savings in implementing the total risk approach in Bangladesh. Cost data presented in this analysis may be used in future cost-effectiveness analyses to compare the total risk and single risk factor approach when considering all costs from a societal perspective to inform health policy in Bangladesh.

The World Health Organization has outlined global targets in the Global Monitoring Framework for the control of NCDs in LMICs, which prioritizes an 80% of availability of affordable basic technologies and essential medicines necessary to treat significant NCDs, including CVDs in both rural and urban areas of the country. Limited CVD treatment access and weak health care infrastructure in Bangladesh is a significant public health concern. As public financing for health care is limited in Bangladesh (~1% of gross domestic product or GDP), public health policies on CVD drug treatment guidelines based on cost estimates, such as out-of-pocket costs is necessary for effective CVD control. Effective policies should address the potential for overtreatment, which comes at a high cost to both the health care system and the patient. The high percentage of the Bangladeshi adult population at low 10-year CVD risk (<10%) highlights the potential for reduction of CVD risk through population-wide public health policy and availability of accessible preventive services. However, caution should be taken to

For peer review only - http://bmjopen.bmj.com/site/about/guidelines.xhtml

ensure that risk stratification approaches are not used in inappropriate clinical circumstances, such as adults with highly uncontrolled hypertension with blood pressure measurements at 160/100 mmHg.

Conclusion

Our data show that the implementation of a total risk approach compared with a single risk factor approach will reduce the health care expenditure by lowering drug costs, which accounts for 60% of out-of-pocket spending in Bangladesh. This approach would be particularly beneficial in Bangladesh, a low-resource country that should prioritize the development of health policy for effective resource allocation in the public health sector. Using the total risk approach would increase service coverage and allow for the distribution of resources to target those at highest risk of experiencing a heart attack or stroke. As the majority of the Bangladeshi adult population aged ≥40 years have a low ten-year risk of CVD, strategies that target those at highest risk of CVD coupled with public health policies to reduce the population-level risk of CVD may be effective.

For peer review only

For peer review only - http://bmjopen.bmj.com/site/about/guidelines.xhtml

Footnotes

Acknowledgments: The study was completed by the National Institute of Ophthalmology (NIO) of Bangladesh with technical assistance from the WHO Country Office for Bangladesh. The authors are grateful to the management and field team of NIO consisting of ophthalmologists, other doctors, nurses, technologists, and enumerators for their hard works.

Competing Interests: The authors of this study declare no conflict of interest. The authors alone are responsible for the views expressed in this article and they do not necessarily represent the views, decisions or policies of the institutions with which they are affiliated.

Funding: Financial assistance for this study was provided by the WHO Country Office for Bangladesh (WHO Reference: 2013/355662-0, Purchase Order: 200843353, Reg. File: BAN-2013-B7-TSA-0001)

Consent to publish: All authors consent to the publication of this manuscript.

Availability of data and materials: The de-identified participant data used and/or analyzed during the current study are available from the corresponding author on reasonable request. Please contact M. Mostafa Zaman at zamanm@who.int for further information and guidelines.

Authors contributions: JYI: conceptualized the manuscript, analyzed data, interpreted results critically, and drafted the manuscript. MMZ: conceptualized the manuscript, designed the study, interpreted results critically, guided manuscript writing, and critically reviewed it. MM, SAS, AHMEH prepared the survey protocol, trained the field team, implemented the survey, processed the data, and reviewed the manuscript. SAS is the guarantor of data.

For peer review only - http://bmjopen.bmj.com/site/about/guidelines.xhtml

Figures:

Figure 1: Prevalence of hypertension, hyperglycemia, and overweight and obesity among Bangladeshi adults aged 40 years and above by area of residence and age group

Figure 2: Proportion of adults with hypertension or severe hypertension by CVD risk group

Figure 3: Annual costs of pharmacologic treatment for CVD by sex and risk stratification approach

For peer review only

For peer review only - http://bmjopen.bmj.com/site/about/guidelines.xhtml

References

1. Checkley W, Ghannem H, Irazola V, Kimaiyo S, Levitt NS, Miranda JJ, et al. Management of NCD in low- and middle-income countries. Global heart. 2014;9(4):431-43. doi: 10.1016/j.gheart.2014.11.003. PubMed PMID: 25592798; PubMed Central PMCID: PMCPMC4299752.

2. Islam SM, Purnat TD, Phuong NT, Mwingira U, Schacht K, Froschl G. Non-communicable diseases (NCDs) in developing countries: a symposium report. Globalization and health. 2014;10:81. doi: 10.1186/s12992-014-0081-9. PubMed PMID: 25498459; PubMed Central PMCID: PMCPMC4267750.

3. Global action plan for the prevention and control of noncommunicable diseases 2013-2020. Geneva, Switzerland: World Health Organization; 2013 [cited 2019 01/10]. Available from: http://apps.who.int/iris/bitstream/10665/94384/1/9789241506236_eng.pdf?ua=1.

4. NCD Global Monitoring Framework: World Health Organization; 2017 [cited 2017 09/29]. Available from: http://www.who.int/nmh/global_monitoring_framework/en/.

5. Prevention of cardiovascular disease: pocket guidelines for assessment and management of cardiovascular disease Geneva: World Health Organization, 2007.

6. Ndindjock R, Gedeon J, Mendis S, Paccaud F, Bovet P. Potential impact of single-risk-factor versus total risk management for the prevention of cardiovascular events in Seychelles. Bulletin of the World Health Organization. 2011;89(4):286-95. doi: 10.2471/BLT.10.082370. PubMed PMID: 21479093; PubMed Central PMCID: PMCPMC3066521.

7. Mendis S, Lindholm LH, Anderson SG, Alwan A, Koju R, Onwubere BJ, et al. Total cardiovascular risk approach to improve efficiency of cardiovascular prevention in resource constrain settings. Journal of clinical epidemiology. 2011;64(12):1451-62. doi: 10.1016/j.jclinepi.2011.02.001. PubMed PMID: 21530172.

For peer review only - http://bmjopen.bmj.com/site/about/guidelines.xhtml

8. Group WCRCW. World Health Organization cardiovascular disease risk charts: revised models to estimate risk in 21 global regions. The Lancet Global health. 2019;7(10):e1332-e45. doi: 10.1016/S2214-109X(19)30318-3. PubMed PMID: 31488387.

9. Fatema K, Zwar NA, Milton AH, Rahman B, Awal AS, Ali L. Cardiovascular risk assessment among rural population: findings from a cohort study in a peripheral region of Bangladesh. Public health. 2016;137:73-80. doi: 10.1016/j.puhe.2016.02.016. PubMed PMID: 27036981.

10. Fatema K, Zwar NA, Milton AH, Rahman B, Ali L. Application of two versions of the WHO/international society of hypertension absolute cardiovascular risk assessment tools in a rural Bangladeshi population. BMJ open. 2015;5(10):e008140. doi: 10.1136/bmjopen-2015-008140. PubMed PMID: 26463220; PubMed Central PMCID: PMCPMC4606419.

11. Fatema K, Rahman B, Zwar NA, Milton AH, Ali L. Short-term predictive ability of selected cardiovascular risk prediction models in a rural Bangladeshi population: a case-cohort study. BMC cardiovascular disorders. 2016;16(1):105. doi: 10.1186/s12872-016-0279-2. PubMed PMID: 27386836; PubMed Central PMCID: PMCPMC4937534.

12. Zaman MM, Rahman MM, Rahman MR, Bhuiyan MR, Karim MN, Chowdhury MA. Prevalence of risk factors for non-communicable diseases in Bangladesh: Results from STEPS survey 2010. Indian journal of public health. 2016;60(1):17-25. doi: 10.4103/0019-557X.177290. PubMed PMID: 26911213; PubMed Central PMCID: PMCPMC6349138.

13. Rahman M, Zaman MM, Islam JY, Chowdhury J, Ahsan HN, Rahman R, et al. Prevalence, treatment patterns, and risk factors of hypertension and pre-hypertension among Bangladeshi adults. Journal of human hypertension. 2018;32(5):334-48. doi: 10.1038/s41371-017-0018-x. PubMed PMID: 29230005; PubMed Central PMCID: PMCPMC5860750.

For peer review only - http://bmjopen.bmj.com/site/about/guidelines.xhtml

For peer review only

14. Karim MN, Zaman MM, Rahman MM, Chowdhury MAJ, Ahsan H, Hassan MM, et al. Sociodemographic Determinants of Low Fruit and Vegetable Consumption Among Bangladeshi Adults: Results From WHO-STEPS Survey 2010. Asia-Pacific journal of public health / Asia-Pacific Academic Consortium for Public Health. 2017;29(3):189-98. doi: 10.1177/1010539517699059. PubMed PMID: 28434246; PubMed Central PMCID: PMCPMC6349139.

15. Surveillance of risk factors for noncommunicable disease: the WHO STEPwise approach. Geneva: World Health Organization, 2001.

16. Population and Housing Census 2001, updated in 2009 Dhaka, Bangladesh: Bangladesh Bureau of Statistics 2009 [cited 2019 01/10]. Available from: http://microdata.worldbank.org/index.php/catalog/1616.

17. Global report on diabetes Geneva, Switzerland: World Health Organization 2016. Available from: https://www.who.int/diabetes/global-report/en/.

18. Mendis S, Lindholm LH, Mancia G, Whitworth J, Alderman M, Lim S, et al. World Health Organization (WHO) and International Society of Hypertension (ISH) risk prediction charts: assessment of cardiovascular risk for prevention and control of cardiovascular disease in low and middle-income countries. Journal of hypertension. 2007;25(8):1578-82. doi: 10.1097/HJH.0b013e3282861fd3. PubMed PMID: 17620952.

19. Population Projection of Bangladesh Dynamics and Trends 2011-2061 Dhaka, Bangladesh Bangladesh Bureau of Statistics 2015 [cited 2018 11/17]. Available from: http://203.112.218.65:8008/WebTestApplication/userfiles/Image/PopMonographs/PopulationProjection.pdf.

20. Khanal MK, Ahmed MS, Moniruzzaman M, Banik PC, Dhungana RR, Bhandari P, et al. Total cardiovascular risk for next 10 years among rural population of Nepal using WHO/ISH risk prediction

For peer review only - http://bmjopen.bmj.com/site/about/guidelines.xhtml

chart. BMC research notes. 2017;10(1):120. doi: 10.1186/s13104-017-2436-9. PubMed PMID: 28270186; PubMed Central PMCID: PMCPMC5341399.

21. Ranawaka UK, Wijekoon CN, Pathmeswaran A, Kasturiratne A, Gunasekera D, Chackrewarthy S, et al. Risk estimates of cardiovascular diseases in a Sri Lankan community. Ceylon Med J. 2016;61(1):11-7. doi: 10.4038/cmj.v61i1.8253. PubMed PMID: 27031973.

22. Ghorpade AG, Shrivastava SR, Kar SS, Sarkar S, Majgi SM, Roy G. Estimation of the cardiovascular risk using World Health Organization/International Society of Hypertension (WHO/ISH) risk prediction charts in a rural population of South India. Int J Health Policy Manag. 2015;4(8):531-6. doi: 10.15171/ijhpm.2015.88. PubMed PMID: 26340393; PubMed Central PMCID: PMCPMC4529043.

23. Otgontuya D, Oum S, Buckley BS, Bonita R. Assessment of total cardiovascular risk using WHO/ISH risk prediction charts in three low and middle income countries in Asia. BMC public health. 2013;13:539. doi: 10.1186/1471-2458-13-539. PubMed PMID: 23734670; PubMed Central PMCID: PMCPMC3679976.

24. Su TT, Amiri M, Mohd Hairi F, Thangiah N, Bulgiba A, Majid HA. Prediction of cardiovascular disease risk among low-income urban dwellers in metropolitan Kuala Lumpur, Malaysia. BioMed research international. 2015;2015:516984. doi: 10.1155/2015/516984. PubMed PMID: 25821810; PubMed Central PMCID: PMCPMC4363497.

25. Vusirikala A, Wekesah F, Kyobutungi C, Oyebode O. Assessment of cardiovascular risk in a slum population in Kenya: use of World Health Organisation/International Society of Hypertension (WHO/ISH) risk prediction charts - secondary analyses of a household survey. BMJ open. 2019;9(9):e029304. doi: 10.1136/bmjopen-2019-029304. PubMed PMID: 31488481; PubMed Central PMCID: PMCPMC6731939.

For peer review only

For peer review only - http://bmjopen.bmj.com/site/about/guidelines.xhtml

26. Cravedi P, Sharma SK, Bravo RF, Islam N, Tchokhonelidze I, Ghimire M, et al. Preventing renal and cardiovascular risk by renal function assessment: insights from a cross-sectional study in low-income countries and the USA. BMJ open. 2012;2(5). doi: 10.1136/bmjopen-2012-001357. PubMed PMID: 23002161; PubMed Central PMCID: PMCPMC3467605.

27. Ahmed MSAM, Moniruzzaman M, Chowdhury S, Banik PC. Cardiovascular Risk Assessment Among Urban Population of Bangladesh Using WHO/ISH Risk Prediction Chart. International journal of epidemiology. 2015;44(suppl_1):i202-i. doi: 10.1093/ije/dyv096.327.

28. Multisectoral Action Plan for the Noncommunicable Disease Control and Prevention (2016-2021) Dhaka, Bangladesh: Ministry of Health and Family Welfare, Government of the People's Republic Bangladesh.; 2015 [cited 2018 12/15]. Available from: http://www.searo.who.int/entity/ncd_tobacco_surveillance/monitoring_fw/pages_from_ban_ncd_action_plan_2016_2021_me_not_approved.pdf.

29. Health and Morbidity Status Survey-2012 Dhaka, Bangladesh: Bangladesh Bureau of Statistics, Ministry of Planning.; 2013 [cited 2018 12/25]. Available from: http://bbs.portal.gov.bd/sites/default/files/files/bbs.portal.gov.bd/page/4c7eb0f0_e780_4686_b546_b4fa0a8889a5/HMSS.pdf.

30. Noncommunicable Diseases Progress Monitor 2017 Geneva, Switzerland: World Health Organization; 2017 [cited 2019 02/15]. 231]. Available from: https://www.who.int/nmh/publications/ncd-progress-monitor-2017/en/.

31. NIPORT. Bangladesh Health Facility Survey 2014 Dhaka, Bangladesh: National Institute of Population Research and Training (NIPORT), Associates for Community and Population Research (ACPR), and ICF International. ; 2016 [cited 2019 03/15]. Available from: https://dhsprogram.com/pubs/pdf/SPA23/SPA23.pdf.

For peer review only - http://bmjopen.bmj.com/site/about/guidelines.xhtml

For peer review only

For peer review only - http://bmjopen.bmj.com/site/about/guidelines.xhtml

Table 1: Background Characteristics of Bangladeshi Adult Participants (n = 6189)

Characteristics	Total (n = 6189)			Men (n = 2824)			Women (n = 3365)		
	Mean (SD)	n	%	Mean (SD)	n	%	Mean (SD)	n	%
Age (years)	52.9 (9.9)			53.5 (10.1)			52.5 (9.8)		
Education (years)	3.1 (4.2)			4.3 (4.8)			2.1 (3.3)		
Area of Residence									
Urban		1873	30.2		808	28.6		1065	31.6
Rural		4316	69.8		2016	71.4		2300	68.4
Occupation									
Professional employment‡		1007	16.3		880	31.3		127	3.8
Industrial worker/Day Laborer		1600	25.9		1440	51.2		160	4.8
Housework		2697	43.7		-	-		2697	80.3
Unemployed/Retired		735	11.9		386	13.7		349	10.4
Other§		131	2.1		106	3.8		25	0.7
Smoking Tobacco Use‖									
Current User		1493	24.2		1432	50.7		61	1.8
Ever User		2261	36.5		2150	76.1		111	3.3
Smokeless Tobacco Use◊									
Current User		3078	49.7		1208	42.8		1870	55.6
Ever User		3469	56.1		1433	50.7		2046	60.5
Alcohol use in the last 30 days									
Yes		75	1.2		68	2.4		7	0.2
Body Mass Index¶	21.9 (4.1)			21.4 (3.7)			22.3 (4.4)		
Waist Circumference** (cm)	82.4 (13.3)			81.8 (10.5)			82.8 (15.2)		
Blood Pressure									
Systolic Blood Pressure††(mmHg)	119.7 (15.2)			119.8 (14.9)			119.7 (15.5)		
Diastolic Blood Pressure‡‡ (mmHg)	80.3 (9.5)			80.2 (9.4)			80.3 (9.7)		
Blood Glucose Levels§§ (mmol/L)	6.9 (3.0)			6.9 (3.0)			6.9 (2.9)		

Abbreviations: SD: Standard Deviation; CM: centimeters; mmHg: millimeter of mercury; mmol/L: millimoles per liter

‡Professional occupation includes: Government employee, private company employee, businessman

§Other occupation includes: Shop keeper, weavers, driver, student, beggar, cook, carpenter, and tailor

‖Excluding smokeless tobacco | Missing values, n = 18; Men, n = 11; Women, n = 7

◊ Smokeless tobacco use includes jorda, white leaf (shaada pata), gul, etc.

¶Body mass index (BMI) calculated by weight in kilogram divided by height in meter squared

For peer review only

For peer review only - http://bmjopen.bmj.com/site/about/guidelines.xhtml

Table 2: Ten-year risk of combined myocardial infarction and stroke (fatal and non-fatal) by gender, using the 2019 WHO CVD Risk non-laboratory based charts for South Asia (n = 5977)

	Total (n = 5977)			Men (n = 2708)			Women (n = 3269)			*P**
	n	%	CI	n	%	CI	n	%	CI	
Very Low Risk (<5%)	3115	52.1	50.8-53.4	1034	38.2	36.6-40.0	2081	63.7	62.0-65.3	<0.001
Low Risk (5-10 %)	1972	33.0	31.8-34.2	1047	38.7	36.8-40.5	925	28.3	26.8-30.0	<0.001
Moderate Risk (10 - <20%)	860	14.4	13.5-15.3	600	22.2	20.6-23.8	260	7.8	7.0-8.9	<0.001
High Risk (20 - <30%)	29	0.5	0.3-0.7	26	1.0	0.6-1.4	3	0.1	0.0-0.2	<0.001
Very High Risk (≥ 30%)	1	0.0	0.0-0.01	1	0.0	0.0-0.1	0	0.0	0.0-0.0	0.272

Abbreviations: CI, confidence interval; WHO, World Health Organization; CVD, Cardiovascular disease

* P-value based on chi-square test

For peer review only

Table 3: Estimation of percentage of population requiring drug treatment based on total risk approach in comparison to single risk factor approaches (n = 5977)

	Cardiovascular Risk ≥ 20%		Single Risk Factor Approach
	CV Risk ≥ 20% alone, %	CV Risk ≥ 20% + BP ≥ 160/100	BP ≥ 140/90 (SBP ≥ 140 + isolated raised DBP), %
Men	1.0	2.1	22.1
Women	0.1	1.6	26.7
Total	0.5	1.8	24.6

Abbreviations: CV, cardiovascular; BP, blood pressure; SBP, systolic blood pressure; DBP, diastolic blood pressure

For peer review only

For peer review only - http://bmjopen.bmj.com/site/about/guidelines.xhtml

Table 4: Population projections and comparison of medicine costs (US$) for implementing total risk approach vs. single risk factor approach in Bangladesh

Estimated annual total cost of CVD medication treatment per million population†

Total risk approach CV Risk ≥ 20% alone

	Total no. of people in the general population (in thousands)*	Percentage of population aged ≥40 years requiring medication (%)	Total no. of people estimated to require drug treatment (in thousands)	No. of people per million population (aged 40 years and older)	Aspirin	Enalapril	Hydrochlorothiazide	Simvastatin	Total
Age Group									
Men									
40 to 49	9210	0.4	3660	4000	$6,570.0	$34,748.0	$12,118.0	$62,196.0	$115,632.0
50 to 59	6303	0.1	596	1000	$1,642.5	$8,687.0	$3,029.5	$15,549.0	$28,908.0
60 to 69	3730	1.5	4733	15000	$24,637.5	$130,305.0	$45,442.5	$233,235.0	$433,620.0
>= 70	1881	7.0	17507	70000	$114,975.0	$608,090.0	$212,065.0	$1,088,430.0	$2,023,560.0
Total	21124	1.0	20769	10000	$16,425.0	$86,870.0	$30,295.0	$155,490.0	$289,080.0
Women									
40 to 49	9087	0.0	0	0	$0.0	$0.0	$0.0	$0.0	$0.0
50 to 59	5662	0.0	0	0	$0.0	$0.0	$0.0	$0.0	$0.0
60 to 69	3257	0.0	0	0	$0.0	$0.0	$0.0	$0.0	$0.0
>= 70	1638	1.6	3965	16000	$26,280.0	$138,992.0	$48,472.0	$248,784.0	$462,528.0
Total	19644	0.1	1958	1000	$1,642.5	$8,687.0	$3,029.5	$15,549.0	$28,908.0
All									
40 to 49	18296	0.2	3605	2000	$3,285.0	$17,374.0	$6,059.0	$31,098.0	$57,816.0
50 to 59	11965	0.1	1134	1000	$1,642.5	$8,687.0	$3,029.5	$15,549.0	$28,908.0
60 to 69	6989	0.7	4206	7000	$11,497.5	$60,809.0	$21,206.5	$108,843.0	$202,356.0
>= 70	3518	4.3	21410	43000	$70,627.5	$373,541.0	$130,268.5	$668,607.0	$1,243,044.0
Total	40768	0.5	20175	5000	$8,212.5	$43,435.0	$15,147.5	$77,745.0	$144,540.0

For peer review only - http://bmjopen.bmj.com/site/about/guidelines.xhtml

Single Risk Factor Approach: BP ≥ 140/90 (SBP ≥ 140 + isolated raised DBP), %

	Total no. of people in the general population (in thousands)*	Percentage of population aged ≥40 years requiring medication (%)	Total no. of people estimated to require drug treatment (in thousands)	No. of people per million population (aged 40 years and older)	Aspirin	Enalapril	Hydrochlorothiazide	Simvastatin	Total
Age Group									
Men									
40 to 49	9210	17.1	156482	171000	$280,867.5	$1,485,477.0	$518,044.5	$2,658,879.0	$4,943,268.0
50 to 59	6303	21.7	129375	217000	$356,422.5	$1,885,079.0	$657,401.5	$3,374,133.0	$6,273,036.0
60 to 69	3730	30.5	96228	305000	$500,962.5	$2,649,535.0	$923,997.5	$4,742,445.0	$8,816,940.0
>= 70	1881	29.4	73529	294000	$482,895.0	$2,553,978.0	$890,673.0	$4,571,406.0	$8,498,952.0
Total	21124	22.1	458995	221000	$362,992.5	$1,919,827.0	$669,519.5	$3,436,329.0	$6,388,668.0
Women									
40 to 49	9087	22.5	199643	225000	$369,562.5	$1,954,575.0	$681,637.5	$3,498,525.0	$6,504,300.0
50 to 59	5662	30.6	164475	306000	$502,605.0	$2,658,222.0	$927,027.0	$4,757,994.0	$8,845,848.0
60 to 69	3257	28.7	81881	287000	$471,397.5	$2,493,169.0	$869,466.5	$4,462,563.0	$8,296,596.0
>= 70	1638	33.7	83509	337000	$553,522.5	$2,927,519.0	$1,020,941.5	$5,240,013.0	$9,741,996.0
Total	19644	26.7	522759	267000	$438,547.5	$2,319,429.0	$808,876.5	$4,151,583.0	$7,718,436.0
All									
40 to 49	18296	20.2	364085	202000	$331,785.0	$1,754,774.0	$611,959.0	$3,140,898.0	$5,839,416.0
50 to 59	11965	26.3	298189	263000	$431,977.5	$2,284,681.0	$796,758.5	$4,089,387.0	$7,602,804.0
60 to 69	6989	29.5	177236	295000	$484,537.5	$2,562,665.0	$893,702.5	$4,586,955.0	$8,527,860.0
>= 70	3518	31.6	157336	316000	$519,030.0	$2,745,092.0	$957,322.0	$4,913,484.0	$9,134,928.0
Total	40768	24.6	992585	246000	$404,055.0	$2,137,002.0	$745,257.0	$3,825,054.0	$7,111,368.0

For peer review only
For peer review only - http://bmjopen.bmj.com/site/about/guidelines.xhtml

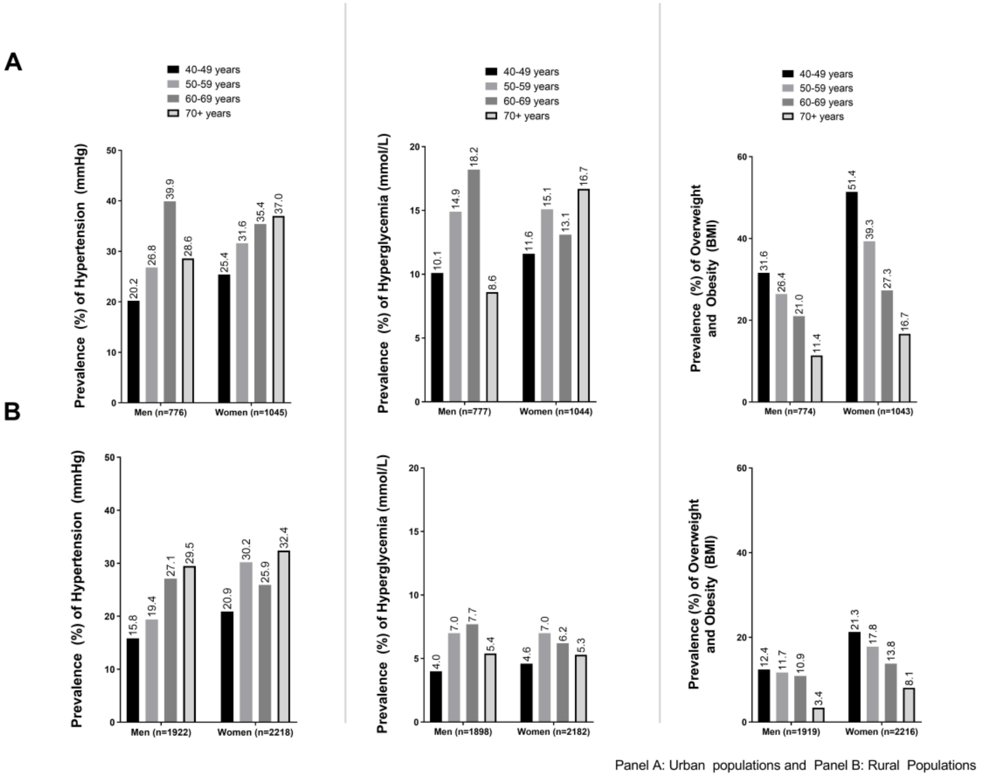

Figure 1: Prevalence of hypertension, hyperglycemia, and overweight and obesity among Bangladeshi adults aged 40 years and above by area of residence and age group.

281x220mm (600 x 600 DPI)

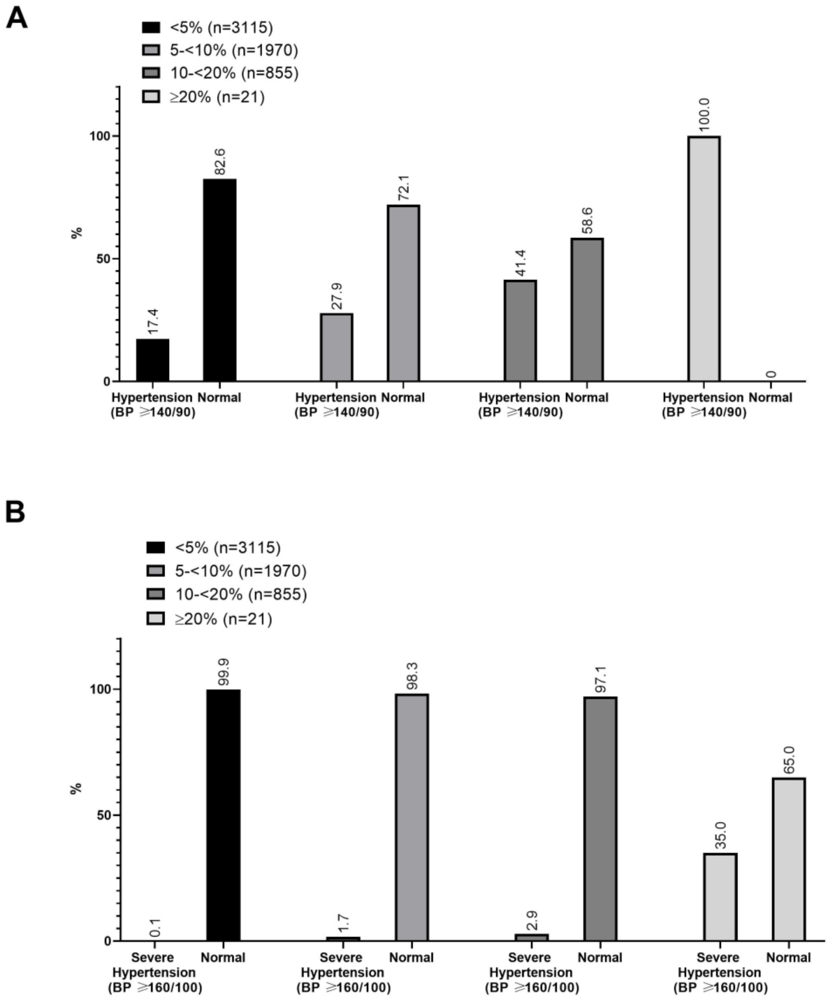

Panel A:% of adults with hypertension by CVD Risk Group
Panel B:% of adults with severe hypertension by CVD Risk Group

Figure 2: Proportion of adults with hypertension or severe hypertension by CVD risk group

205x276mm (300 x 300 DPI)

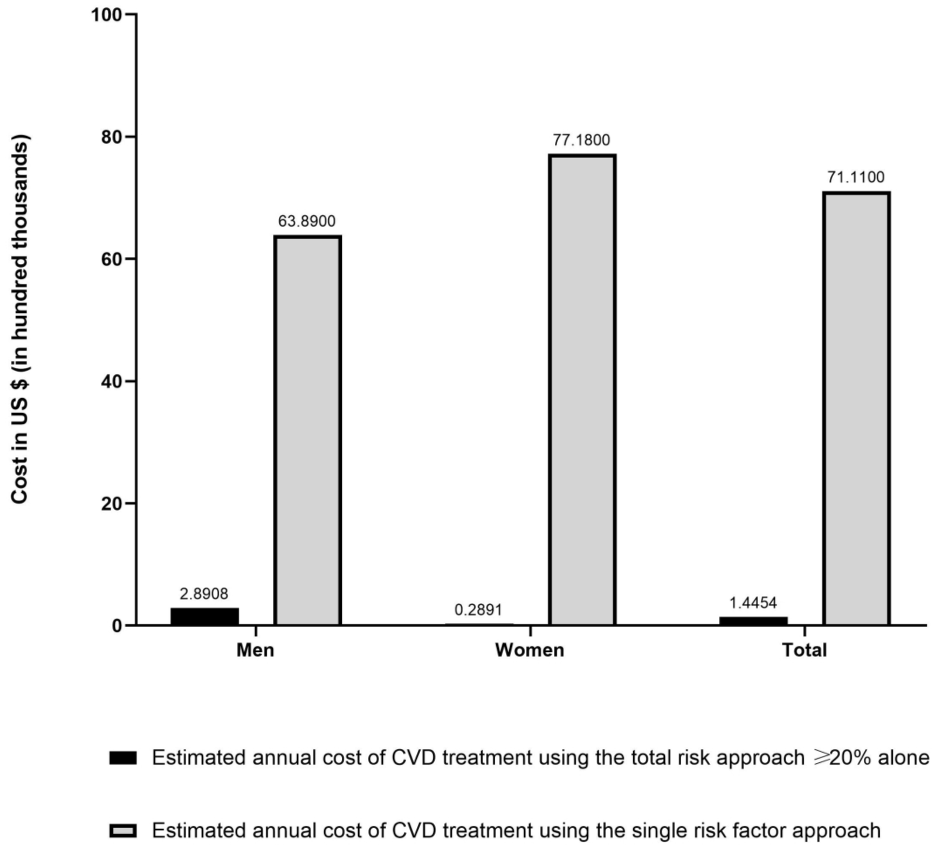

Figure 3: Annual costs of pharmacologic treatment for CVD by sex and risk stratification approach

199x175mm (300 x 300 DPI)

Appendix Table 1: Cost of common drugs used to treat cardiovascular disease in Bangladesh (converted to US$)

Drug Name	Dose	Number of Tablets	Category	Median price (in Bangladeshi Taka)	Price (in US$)*
Aspirin	100 mg	100 tablets	Antiplatelet drugs	38BDT	0.45
Atenolol	50 mg	100 tablets	Antihypertensive, Beta-adrenoreceptor blocking drugs	77BDT	0.92
Captopril	25 mg	100 tablets	Antihypertensive, ACE inhibitors	300 BDT	3.58
Chrlorthalidone	25 mg	30 tablets	Antihypertensive, thiazide diuretics	60 BDT	0.72
Enalapril	10 mg	100 tablets	Antihypertensive, ACE inhibitors	200 BDT	2.38
Frusemide	40 mg	100 tablets	Antihypertensive, loop diuretics	53 BDT	0.63
Hydrochlorothiazide	25 mg	100 tablets	Antihypertensive, thiazide diuretics	70 BDT	0.83
Isosorbide dinitrate	10 mg	100 tablets	Angina treatment, nitrates - coronary vasodilators	35 BDT	0.42
Labetalol	100 mg	60 tablets	Antihypertensive, Beta-adrenoreceptor blocking drugs	361.2 BDT	4.31
Losartan	50 mg	100 tablets	Antihypertensive, Angiotensin-II receptor blocker	600 BDT	7.15
Lovastatine	20 mg	30 tablets	Lipid regulation, statin	303.3 BDT	3.62
Methyldopa	250 mg	100 tables	Antihypertensive, Centrally acting or central sympatholytic	308 BDT	3.67
Nifedipine (slow release)	20 mg	100 tablets	Angina treatment, calcium-channel blocker	64 BDT	0.76
Propranolol hydrochloride	10 mg	100 tablets	Antihypertensive, Beta-adrenoreceptor blocking drugs	24 BDT	0.29
Simvastatine	10 mg	30 tablets	Lipid regulation, statin	360 BDT	4.29

For peer review only

Spironolactone	25 mg	100 tablets	Antihypertensive, potassium-sparing diuretics & aldoesterone antagonist	202 BDT	2.41
Streptokinase	1.5 million unit/vial	One vial	Anticoagulant	3100 BDT	36.96
Warfarin	5 mg	100 tablets	Anticoagulant	300 BDT	3.58

Abbreviations: mg, milligrams; US, United States; BDT, Bangladeshi Taka

*Price conversion based on exchange rate on 02/07/2019

For peer review only

STROBE 2007 (v4) Statement—Checklist of items that should be included in reports of *cross-sectional studies*

Section/Topic	Item #	Recommendation	Reported on page #
**Title and abstract**	1	(*a*) Indicate the study's design with a commonly used term in the title or the abstract	2
		(*b*) Provide in the abstract an informative and balanced summary of what was done and what was found	2-3
**Introduction**			
Background/rationale	2	Explain the scientific background and rationale for the investigation being reported	5-6
Objectives	3	State specific objectives, including any prespecified hypotheses	6
**Methods**			
Study design	4	Present key elements of study design early in the paper	6
Setting	5	Describe the setting, locations, and relevant dates, including periods of recruitment, exposure, follow-up, and data collection	7
Participants	6	(*a*) Give the eligibility criteria, and the sources and methods of selection of participants	6
Variables	7	Clearly define all outcomes, exposures, predictors, potential confounders, and effect modifiers. Give diagnostic criteria, if applicable	8-10
Data sources/ measurement	8*	For each variable of interest, give sources of data and details of methods of assessment (measurement). Describe comparability of assessment methods if there is more than one group	7-10
Bias	9	Describe any efforts to address potential sources of bias	6-7 & 15-16
Study size	10	Explain how the study size was arrived at	7
Quantitative variables	11	Explain how quantitative variables were handled in the analyses. If applicable, describe which groupings were chosen and why	9-10
Statistical methods	12	(*a*) Describe all statistical methods, including those used to control for confounding	9-10
		(*b*) Describe any methods used to examine subgroups and interactions	8-11
		(*c*) Explain how missing data were addressed	9
		(*d*) If applicable, describe analytical methods taking account of sampling strategy	N/A
		(*e*) Describe any sensitivity analyses	N/A
**Results**			

For peer review only

Participants	13*	(a) Report numbers of individuals at each stage of study—eg numbers potentially eligible, examined for eligibility, confirmed eligible, included in the study, completing follow-up, and analysed	10
		(b) Give reasons for non-participation at each stage	N/A
		(c) Consider use of a flow diagram	N/A
Descriptive data	14*	(a) Give characteristics of study participants (eg demographic, clinical, social) and information on exposures and potential confounders	10
		(b) Indicate number of participants with missing data for each variable of interest	9
Outcome data	15*	Report numbers of outcome events or summary measures	
Main results	16	(*a*) Give unadjusted estimates and, if applicable, confounder-adjusted estimates and their precision (eg, 95% confidence interval). Make clear which confounders were adjusted for and why they were included	N/A
		(*b*) Report category boundaries when continuous variables were categorized	N/A
		(*c*) If relevant, consider translating estimates of relative risk into absolute risk for a meaningful time period	N/A
Other analyses	17	Report other analyses done—eg analyses of subgroups and interactions, and sensitivity analyses	10
**Discussion**			
Key results	18	Summarise key results with reference to study objectives	12-14
Limitations	19	Discuss limitations of the study, taking into account sources of5potential bias or imprecision. Discuss both direction and magnitude of any potential bias	15-16
Interpretation	20	Give a cautious overall interpretation of results considering objectives, limitations, multiplicity of analyses, results from similar studies, and other relevant evidence	13-16
Generalisability	21	Discuss the generalisability (external validity) of the study results	15
**Other information**			
Funding	22	Give the source of funding and the role of the funders for the present study and, if applicable, for the original study on which the present article is based	19

*Give information separately for cases and controls in case-control studies and, if applicable, for exposed and unexposed groups in cohort and cross-sectional studies.

Note: An Explanation and Elaboration article discusses each checklist item and gives methodological background and published examples of transparent reporting. The STROBE checklist is best used in conjunction with this article (freely available on the Web sites of PLoS Medicine at http://www.plosmedicine.org/, Annals of Internal Medicine at http://www.annals.org/, and Epidemiology at http://www.epidem.com/). Information on the STROBE Initiative is available at www.strobe-statement.org.

For peer review only
